# Promoter-level expression clustering identifies time development of transcriptional regulatory cascades initiated by ErbB receptors in breast cancer cells

**DOI:** 10.1038/srep11999

**Published:** 2015-07-16

**Authors:** Marco Mina, Shigeyuki Magi, Giuseppe Jurman, Masayoshi Itoh, Hideya Kawaji, Timo Lassmann, Erik Arner, Alistair R. R. Forrest, Piero Carninci, Yoshihide Hayashizaki, Carsten O. Daub, the FANTOM Consortium, Mariko Okada-Hatakeyama, Cesare Furlanello

**Affiliations:** 1 Fondazione Bruno Kessler, Via Sommarive 18, I-38123 Povo, Trento, Italy; 2 Laboratory for Integrated Cellular Systems, RIKEN Center for Integrative Medical Sciences (IMS), Tsurumi-ku, Yokohama, Kanagawa 230-0045, Japan; 3 RIKEN Center for Life Science Technologies (Division of Genomic Technologies) (CLST (DGT)), 1-7-22 Suehiro-cho, Tsurumi-ku, Yokohama, Kanagawa 230-0045, Japan; 4 RIKEN Omics Science Center (OSC), 1-7-22 Suehiro-cho, Tsurumi-ku, Yokohama 230-0045, Japan; 5 RIKEN Preventive Medicine and Diagnosis Innovation Program (PMI), 2-1 Hirosawa, Wako-shi, Saitama 351-0198, Japan; 6 Telethon Kids Institute, The University of Western Australia, 100 Roberts Road, Subiaco, WA 6008, Australia; 7 Department of Biosciences and Nutrition and Science for Life Laboratory, Karolinska Institutet, Stockholm, SE-141 86, Sweden; 8 Department of Medicine at Karolinska Institutet and Center for Metabolism and Endocrinology at Karolinska University Hospital, 141 86, Stockholm, Sweden

## Abstract

The analysis of CAGE (Cap Analysis of Gene Expression) time-course has been proposed by the FANTOM5 Consortium to extend the understanding of the sequence of events facilitating cell state transition at the level of promoter regulation. To identify the most prominent transcriptional regulations induced by growth factors in human breast cancer, we apply here the Complexity Invariant Dynamic Time Warping motif EnRichment (CIDER) analysis approach to the CAGE time-course datasets of MCF-7 cells stimulated by epidermal growth factor (EGF) or heregulin (HRG). We identify a multi-level cascade of regulations rooted by the Serum Response Factor (SRF) transcription factor, connecting the MAPK-mediated transduction of the HRG stimulus to the negative regulation of the MAPK pathway by the members of the DUSP family phosphatases. The finding confirms the known primary role of FOS and FOSL1, members of AP-1 family, in shaping gene expression in response to HRG induction. Moreover, we identify a new potential regulation of DUSP5 and RARA (known to antagonize the transcriptional regulation induced by the estrogen receptors) by the activity of the AP-1 complex, specific to HRG response. The results indicate that a divergence in AP-1 regulation determines cellular changes of breast cancer cells stimulated by ErbB receptors.

Upon stimulation by growth factors, ErbB receptors trigger signals that propagate into the cytosol by recruiting the cascades of protein kinases known as Akt and MAPK (ERK, JNK and p38 MAPK) signaling pathways^1^. The signal is propagated by sequential phosphorylation to the nucleus to modulate the activity of regulatory molecules, such as ELK1 and the Serum Response Factor (SRF), eventually leading to cellular changes^2^. It has been shown that activation, magnitude, duration and subsequent inactivation of the MAPK signalling pathway during the cellular response to external cues induce the appropriate changes required for the determination of cell fate^1^,^3^. Not surprisingly, the deregulation of ErbB receptors is associated to a variety of human cancers^4^.

In the MCF-7 breast cancer cell line, the ErbB receptor ligand heregulin (HRG) induces a sustained MAPK signal activity that triggers an irreversible cell phenotype shift toward differentiation (accumulation of lipid droplets within the cells). On the contrary, the EGF receptor (EGFR) ligand EGF only elicits a transient MAPK signal activity and stimulates cell proliferation^5^,^6^. The sustained or transient activity patterns of the same MAPK signalling pathway, respectively induced by EGF and HRG, elicit an all-or-none response in activation of Transcription Factors (TFs)^7^, which eventually drives the cell toward proliferation or differentiation, respectively. For example, only a sustained ERK activity can stabilize the newly synthesized proteins of immediate early gene (IEG), such as FOS, FOSL and MYC^8^, involved in the mechanisms of cell fate control.

The identification of the transcriptional networks initiated by ErbB receptors under different conditions is important to characterize the early phase of cell proliferation and differentiation, and to understand cancer progression and prevention. Previous works based on microarray data provided important insights on the downstream targets of ERK activation in MCF-7 cells, identifying the TFs that are activated and the corresponding induced genes^5^,^6^. Among the transcription factors known to be induced by ERK1/2 activation, SRF is of primary importance since it is involved in the mechanisms of cell type differentiation and response to physiological and environmental stimuli. A central problem is to understand how SRF activity can be differentially controlled according to cell type and signalling pathway^9^.

The analysis of CAGE (Cap Analysis of Gene Expression) time-courses has been applied to examine the dynamics of enhancer and promoter activities, in order to extend the understanding of the sequence of events facilitating cell state transition by sequentially analyzing transcription start sites^10^. To extend the understanding of the time-dependent transcriptional regulatory cascade triggered in MCF-7 cells by ErbB receptor activation, we studied the CAGE time-course data for growth factor-stimulated MCF-7 breast cancer cells. We applied the Complexity Invariant Dynamic Time Warping motif EnRichment (CIDER) method^11^ to the whole MCF-7 CAGE time-course data to infer the prominent regulatory mechanisms and the respective driving TFs. Our analysis allows reconstructing the regulatory chain induced by ErbB pathway activation through the induction of SRF, moving from the action of immediate early genes to the effect of late TFs on late time-points. The results largely confirm the observations from previous works, elucidate some mechanisms not completely observed before, and propose new putative regulations.

This work is part of the FANTOM5 project. Data downloads, genomic tools and co-published manuscripts are summarized here: http://fantom.gsc.riken.jp/5/.

## Results

### Differential activation dynamics of upstream signals triggered by EGF and HRG stimulation

**Figure 1:**
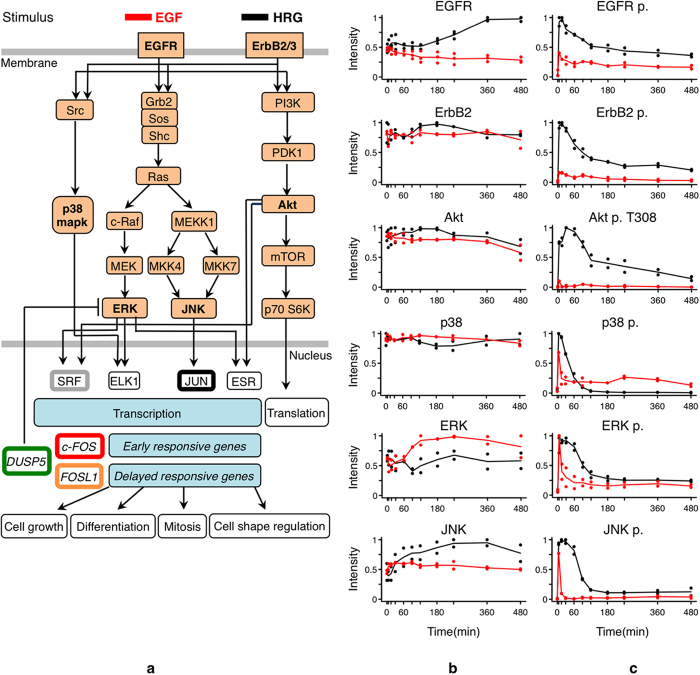
(**a**) The signal transduction pathway activated upon stimulation of MCF-7 cells by HRG or EGF. (**b**) Comparison of MAPK proteins abundance after the stimulation of the same ligands (10 nM) by HRG (black pattern) and EGF (red pattern), quantified by Western blot analysis. (**c**) Comparison of MAPK proteins phosphorylation levels after the stimulation of the same ligands (10 nM) by HRG (black pattern) and EGF (red pattern), quantified by Western blot analysis. Western blot experiments were performed twice. Each dot in panels a and b represents the quantified values in an independent experiment, and lines represent the average of these values.

To confirm the series of biochemical events initiated by HRG and EGF, we first examined by Western blot analysis the activation of the MAPK and Akt signaling pathways in MCF-7 cells at 0 min (non-treated), 15 min, 30 min, 45 min, 60 min, 80 min, 100 min, 2 hr, 2.5 hr, 3 hr, 3.5 hr, 4 hr, 5 hr, 6 hr, 7 hr and 8 hr after EGF or HRG stimulation (Fig. 1). Both EGF and HRG induced early peaks of signal response within 30 mins after ligand stimulation. HRG induced strong and persistent activation (phosphorylation) in EGFR, ErbB2, ERK, JNK, p38 MAPK and Akt, while EGF induced only weak (EGFR, ErbB2 and Akt) and transient (ERK, JNK and p38 MAPK) signals (Fig. 1b,c, Supplementary Figures S1-S2).

**Figure 2:**
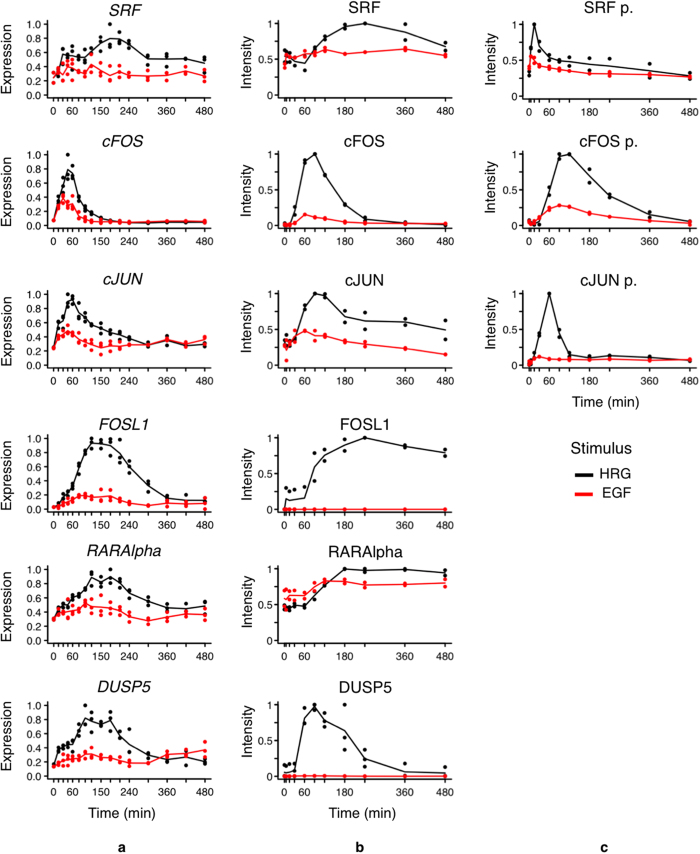
Characterization of SRF, cFOS, cJUN, FOSL1, RARAlpha and DUSP5 genes at expression and protein levels. (**a**) CAGE analysis captures the quantitative differences in transcriptional changes induced by EGF (red)- and HRG (black)-stimulated signaling pathways in MCF-7 cells. (**b**) Western blot analysis was performed to quantify protein levels after the stimulation of the same ligands (10 nM). (**c**) Western blot analysis was performed to quantify protein phosphorylation levels after the stimulation of the same ligands (10 nM). Western blot experiments were performed twice. Each dot in panels b and c represents the quantified values in an independent experiment, and lines represent the average of these values.

Consistently with such signaling pathway activation patterns, the CAGE analysis showed expression peaks for the IEGs SRF, FOS and JUN, known to be activated downstream of MAPK activation, with higher expression levels in the HRG-stimulated cells compared to EGF-stimulation (Fig. 2a). We verified by Western blot analysis that the higher expression of SRF, FOS and JUN in HRG-treated cells is followed by higher protein and phosphorylation (activation) levels (Fig. 2b,c, Supplementary Figures S1-S2). Overall, the quantitative time-course trends are very similar between CAGE and Western blot-based proteomic results.

### HRG and EGF ligands induce regulatory programs sharing a core of early regulatory interactions

**Figure 3:**
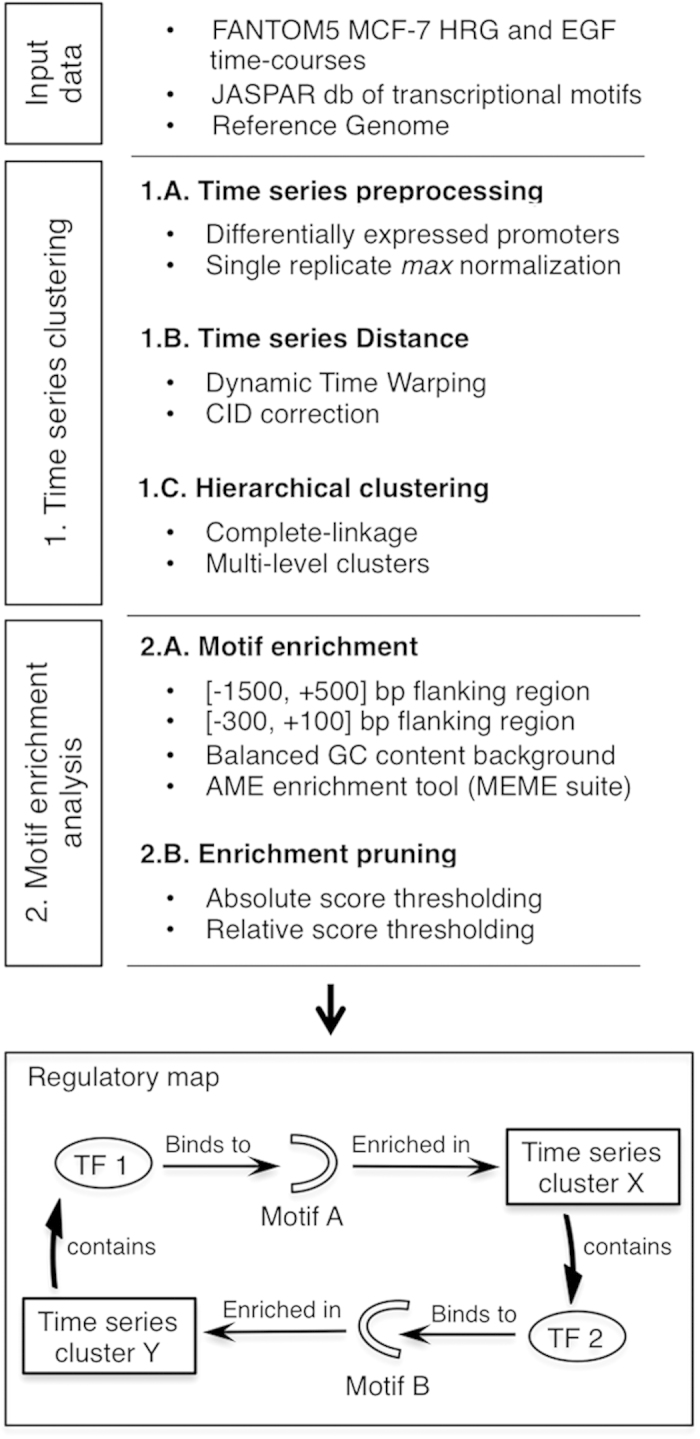


**Figure 4:**
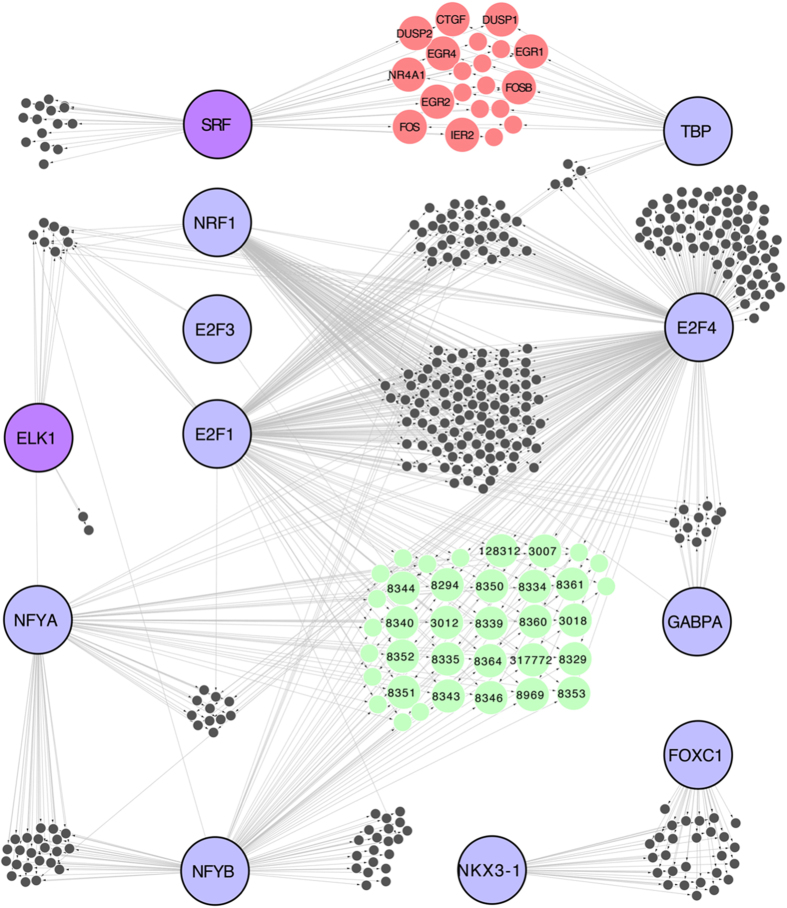
Intersection of the regulatory maps inferred by CIDER for the MCF-7 HRG- and EGF-induced time-courses. This network of 800 predicted interactions associates 12 TFs (large blue/pink nodes) to 386 target promoters (gray, pale green and red nodes). The cluster of 36 pale green nodes includes 30 genes coding for Histone proteins (Supplementary Data 4). The cluster of red nodes includes the FOS TF.

We applied CIDER to the CAGE time-course data obtained from MCF-7 cells stimulated with HRG and EGF (Fig. 3), in order to infer the stimulus-specific transcriptional waves induced in the MCF-7 regulatory network. CIDER analysis inferred 8470 putative regulatory interactions between 35 TFs and 2575 promoters for the HRG time-course, and 3968 regulatory interactions between 19 TFs and 988 promoters for the EGF time-course. The complete lists of interactions for the HRG and EGF time-courses are available as Supplementary Data 1 and 2, respectively. In order to highlight the commonalities between the regulatory programs respectively induced by EGF and HRG, we considered the intersection of the two regulatory maps, consisting of a shared core of 800 predicted interactions associating the activity of 12 TFs to 386 target promoters (Fig. 4, Supplementary Data 3). This core network is structured in clusters of promoters co-regulated by specific sets of TFs, in which the major role is played by seven TFs (E2F1, E2F3, E2F4, FOXC1, NFYA, NKX31, SRF) involved in the regulation of cell proliferation. We observe that NFYA/B, E2F1 and E2F4 TFs, which orchestrate the largest component of the network (322 nodes), co-regulate a cluster of 36 promoters (green nodes in Fig. 4) partially mapping to histone genes (the cluster is enriched for the *nucleosome assembly* Gene Ontology (GO) term, enrichment P value: 7.76 10^−10^, see Supplementary Data 4). Interestingly, the EGF and HRG time-courses share the regulatory activity of SRF and ELK1 TFs, known to be activated downstream of the MAPK pathway (Fig. 1a). The cluster of genes regulated by SRF (red nodes in Fig. 4) is enriched for *Response to stimulus* (GO enrichment P value 6.53 10^−06^, list of genes in Supplementary Table S1), and includes the FOS TF.

In the following sections we specifically focus on the regulatory network downstream of the SRF and FOS regulation, as FOS is known to be activated downstream of MAPK activation and is vital in controlling the ERK signaling pathway for MCF-7 cell differentiation^5^.

### SRF starts a complex regulatory cascade in response to HRG stimulation

**Figure 5:**
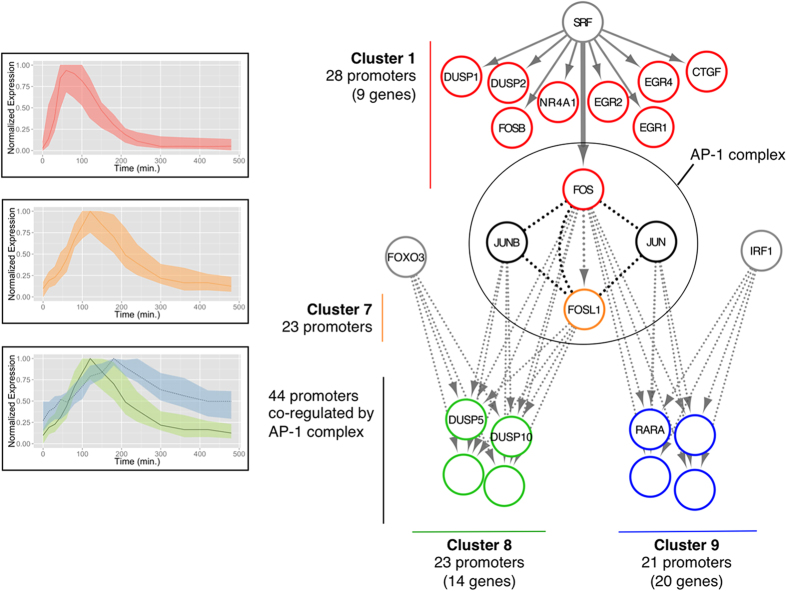
The SRF-rooted regulatory sub-network of MCF-7 cells in response to HRG- and EGF-induction. Solid gray edges indicate the transcriptional regulations inferred by CIDER for both HRG and EGF time-courses. Dashed gray edges indicate the transcriptional regulations inferred by CIDER only for the HRG time-course. The interplay between JUN, JUNB, FOS and FOSL1 in the AP-1 complex at protein level is represented by the undirected black lines. The plots on the left show the expression patterns of the four clusters 1 (red), 7 (orange), 8 (green) and 9 (blue).

By following the SRF-rooted transcriptional cascade within the transcriptional network inferred by CIDER, we unveiled a complex regulatory mechanism in the HRG time-course (Fig. 5). The transcriptional program connects the activity of the MAPK pathway to a multitude of functional components, including four phosphatases (DUSP1/2/5/10) involved in the negative regulation of the MAPK pathway. According to CIDER analysis, only the first portion of this regulatory cascade, corresponding to the direct regulatory activity of SRF, is active in the EGF time-course, while the downstream regulatory events are HRG-specific, with the missing activity of the Activator Protein 1 (AP-1) TF in the EGF time-course (Fig. 5). This may be because the HRG-induced sustained and strong ERK activity has more significant impact in gene expression and protein stabilization of downstream transcription factors than EGF-induced transient kinase activity^6^,^7^. The observed differential regulation between HRG and EGF induction is consistent with the depleted expression of the targeted genes in the EGF time-course, compared to HRG, as discussed in the next sections. The sets of inferred interactions for the HRG and EGF cascades initiated by SRF are provided in Supplementary Data 5 and 6, respectively. To empirically estimate the False Discovery Rate (FDR) of the predictions, we determined which fraction of the 24 SRF interactions inferred for either the EGF or HRG time-courses is supported by Chromatin Immunoprecipitation (ChIPSeq) data from the ENCODE project^12^ (see Methods). Overall, we found peaks of SRF binding activity supporting 16 interactions (FDR: 33%, Supplementary Table S1). The FDR improves to 20% when considering only the 10 interactions shared by the EGF and HRG cascades.

### SRF leads the immediate early response in both HRG and EGF time-courses

**Figure 6:**
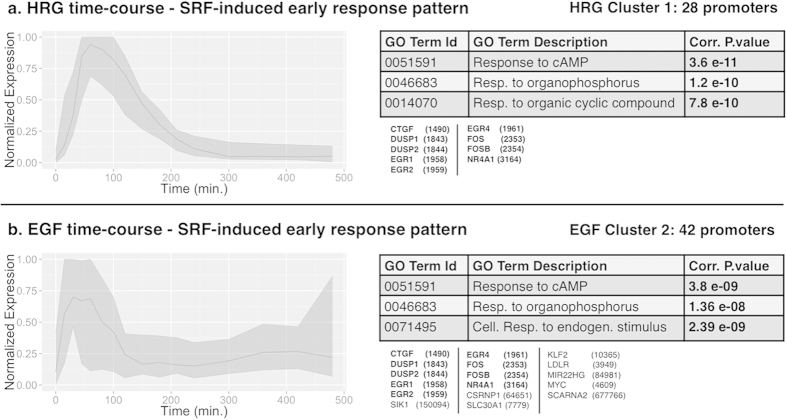
The expression patterns of the HRG Cluster 1 (**a**) and the EGF Cluster 2 (**b**), enriched for the Jaspar MA0476.1 motif (SRF transcription factor). Left: representation of the average expression pattern (lines) and 10%–90% quantiles interval (colored regions). Right: selection of Gene Ontology terms most enriched (top tables) and list of genes included in the clusters (gene names and Entrez ids).

For the HRG time-course, CIDER associated SRF to the regulation of a cluster of 28 promoters (9 genes), hereby referred to as Cluster 1, characterized by an early response pattern (Fig. 6a). Cluster 1 includes the *FOS*, *DUSP1/2*, *EGR1/2/4*, and *NR4A1* early genes, and is enriched for multiple *response to stimulus* Gene Ontology terms (GO enrichment P value 6.53 10^−06^, gene list in Supplementary Data 5). The FOS transcription factor is a typical IEG induced by activation of ERK signal^13^. *DUSP1* expression has been reported to show rapid increase in early time points, following ERK activation^14^. EGR4 is a zinc-finger protein, a member of the EGR family of transcription regulatory factors that plays a critical role in mediating enduring forms of neuronal plasticity and in the regulation of inflammatory cytokine gene transcription^15^. The SRF-mediated regulation of these transcription factors seems to be rather common and conserved regardless of different cell types. Indeed, the involvement of SRF inferred by CIDER in the regulation of this cluster is consistent with the results of a similar study on hippocampal rat cells^16^, where a time-series cluster analysis of a set of 33 genes identified a subset of genes, including *FOS*, *EGR1*, *EGR2*, *EGR4*, *DUSP1* and *NR4A1*, enriched for the SRF binding motif. Chromatin Immunoprecipitation (ChIP) experiments confirmed that SRF was binding to the promoters of *FOS*, *EGR1*, *EGR2* and *NR4A1*^16^. Moreover, it is known that the expression of *FOS*, *EGR1* and *EGR2* is regulated by SRF in various plasticity models^17^,^18^,^19^,^20^,^21^,^22^. The experimental evidence available in literature supporting the set of inferred interactions for the SRF cascade is collected in Table 1. The interactions were also verified *in silico* with the LASAGNA web-service^23^. We further validated the SRF binding activity on the nine genes of Cluster 1 using the ENCODE ChIPSeq dataset, finding support for all the interactions but DUSP1 and CTGF (Supplementary Table S1). These results are consistent with those obtained by Motif Activity Response Analysis (MARA), the transcriptional inference methodology used in the FANTOM Consortium^24^: six of the nine genes in Cluster 1 (NR4A1, FOSB, EGR1, EGR2, EGR4, DUSP2) were predicted as downstream targets of SRF by MARA (Fisher’s test P value: 4.70 e^−10^, Supplementary Table S2). The comparison of CIDER and MARA analysis on the MCF-7 HRG time-course is detailed in the Supplementary Material.

The SRF regulation of the nine genes included in Cluster 1 was inferred by CIDER also for the EGF time-course, where SRF was associated to the regulation of a cluster of 42 promoters (17 genes), hereby referred to as Cluster 2 (Fig. 6b, gene list included in Supplementary Table 1 and Supplementary Data 6). This finding suggests that the immediate phase of transcription by SRF is qualitatively similar between HRG- and EGF-induction. Consistently with the early expression patterns of Clusters 1 and 2, the SRF phosphorylation, required for SRF binding activity^25^, rapidly increases in the first time-points, reaching its maximum at 15 and 30 min after induction, respectively for the EGF and the HRG case (Fig. 2c). As SRF phosphorylation is stronger in the HRG case, the expression of genes in the HRG Cluster 1 is significantly prolonged and stronger than the EGF Cluster 2 (Fig. 6, FOS expression is detailed in Fig. 2a). Note that CIDER found a second SRF enriched cluster for the HRG time-course (Cluster 3), and three additional clusters (Clusters 4–6) were enriched for SRF only in less stringent definition of the flanking regions [–1500, +500] bp (Supplementary Material).

To summarize, we assist to a qualitatively similar, but quantitatively different, immediate response downstream of the ErbB2 pathway between the HRG and EGF time-courses at the transcriptional level, since the early phase of induction.

### FOS and FOSL1 play a central role in the HRG-induced transcriptional network

**Figure 7:**
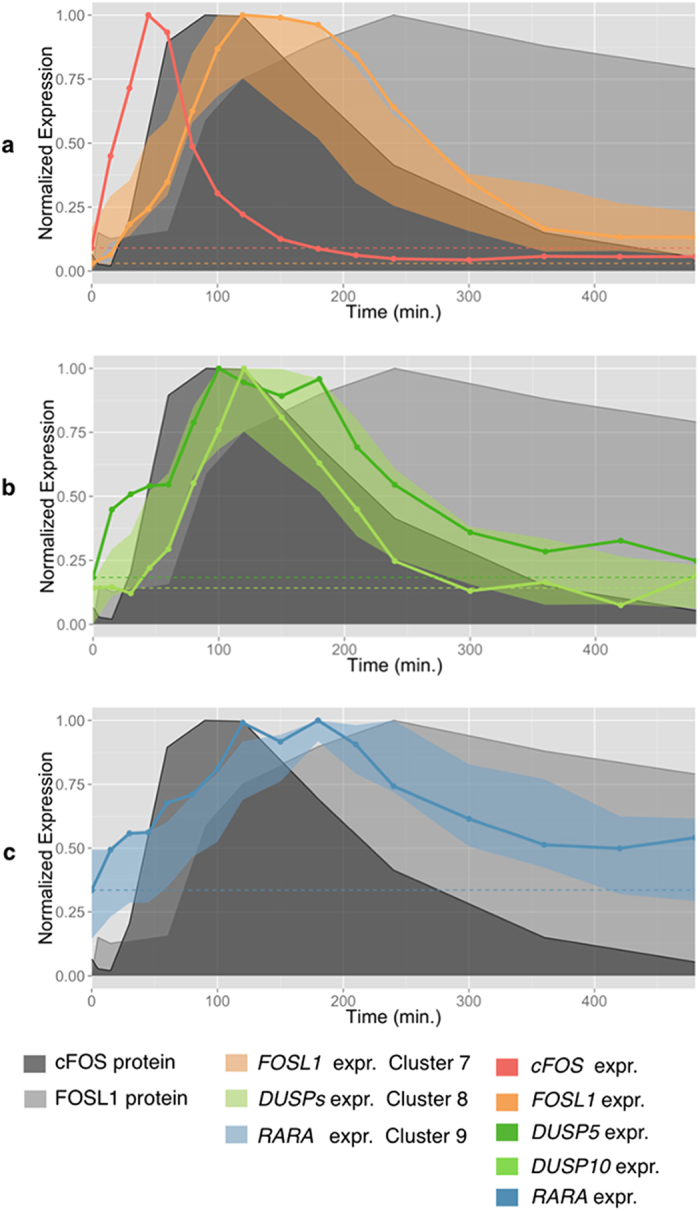
(**a**) FOSL1 regulation by FOS in HRG-induced MCF-7 cells. cFOS expression (red curve) and protein abundance (dark gray region) rapidly increase after HRG-induction. Consistently, *FOSL1* expression (orange curve) increases in the 45–120 min time span, and decreases as FOS protein abundance decreases. FOSL1 protein level, instead, is sustained up to 8 hrs. (**b**) DUSP5/DUSP10 regulation by AP-1 complex. DUSP5/10 expression (green curves) increases in the 45–120 min time span, and decreases as FOS protein abundance decreases. (**c**) RARA regulation by AP-1 complex. RARA expression (blue curve) increases peaks in the 100–180 min time interval, and slowly decreases at late time-points.

Downstream of the *FOS* induction by SRF in the HRG time-course, CIDER unveiled a complex regulatory mechanism centered on the activity of the four TFs FOS, FOSL1, JUN and JUNB (Fig. 5, Supplementary Data 5). These TFs are known to interact in the Activator Protein 1 (AP-1), a nuclear transcription factor comprising homodimers and heterodimers of members of the FOS family (*c-FOS, FOSB, FRA-1/FOSL-1* and *FRA-2/FOSL-2*)^26^,^27^,^28^,^29^,^30^ and Jun family (*c-JUN, JUNB* and *JUND*)^31^,^32^,^33^,^34^. The mechanisms within the AP-1 complex have not been fully elucidated yet, but it is known that FOS proteins cannot form stable homodimers alone^35^, suggesting that FOS and FOSL1 interact with each other. In addition, FOS and FOSL1 also interact with other AP-1 partners, such as JUN protein^5^. Both CAGE and proteome analysis (Fig. 2) clearly showed expression of JUN; therefore, there might exist a modulatory protein-protein interaction between JUN and FOS/FOSL1 (Fig. 5, black edges). The analysis suggests that *FOSL1* is transcriptionally regulated by *FOS* (Cluster 7, genes listed in Supplementary Data 5); *FOS* and *FOSL1* expression is almost absent before HRG-induction, and it rapidly increases after induction, peaking at 45 min and in the interval 2 h-2 h30 min, respectively (Fig. 2a). *FOSL1* expression mimics the FOS protein level with a slight time delay (Fig. 7a), confirming the transcriptional dependence of FOSL1 from FOS. Consistently, the regulation of *FOSL1* by *FOS* has been confirmed in multiple works^36^,^37^,^38^,^39^.

CIDER inferred a combined activity of the AP-1 complex on two clusters of 23 (Cluster 8) and 21 promoters (Cluster 9), characterized by different expression patterns (Fig. 7b,c), suggesting the existence of two different regulatory mechanisms (genes listed in Supplementary Data 5). Remarkably, we did not find any regulatory interaction between *FOS* and *FOSL1*, nor any involvement of FOSL1 in the regulatory network, when analyzing the EGF-induced time-course. Indeed, in the EGF-induced time-course the expression of *FOS* reaches only half of the levels registered in the HRG case, while *FOSL1* is almost depleted (Fig. 2a). Our CAGE peak expression and proteomic analyses indicate that the EGF-induced lower expression of *FOS* mRNA, combined with transient ERK activity, result in lowered FOS protein expression (Fig. 2b), and they are major factors for the depleted expression of *FOSL1*. Consistently with these results, the FOSL1 protein level in HRG is evident, while there is no significant level of FOSL1 in EGF-induced time-course (Fig. 2b).

### Transcriptional regulation of FOS and FOSL1 in early HRG response

Cluster 8 has an early and transient expression that follows the pattern of FOS protein, peaking at 100 minutes and decreasing back to basal levels within 6 hours after induction (Fig. 7b). The level of FOSL1 protein is sustained at late time points, suggesting that FOSL1 might have a negative regulatory effect on the promoters in Cluster 8. The Gene Ontology enrichment analysis found the cluster enriched for the *MAP kinase tyrosine/serine/threonine phosphatase activity* molecular function (corrected P value: 3.6 10^−4^). MAP kinase phosphatases (MKPs) constitute a family of dual-specificity phosphatases (DUSP) that inactivate the MAPKs by dephosphorylating both the phospho-Thr and phospho-Tyr regulatory residues^14^,^36^, and have been related to the development of many types of cancer and to the outcome of different chemotherapeutic treatments^40^. For example, DUSPs have been found to be less expressed in several types of cancer^41^. As described earlier, our analysis identified *DUSP1* as direct target of SRF, thus explaining part of the negative feedback mechanism triggered by the MAPK (p38, JNK and ERK) activation by HRG, and leading to the re-establishment of the basal level of MAPK activity. Similarly, breast carcinomas have reduced expression of the ERK1/2-specific phosphatase DUSP5^42^. In addition, JUN regulates DUSP5 expression through the AP-1 complex, in response to ERK pathway activation, delineating a negative feedback mechanism^36^. Consistently with the current literature and the LASAGNA web-service, we found that DUSP5 and DUSP10 are members of Cluster 8, regulated by the AP-1 complex (Figs 5 and 7b).

Within the regulatory map inferred by CIDER, Cluster 8 is also selectively enriched for the FOXO3 motifs (Cluster 9 is not enriched for this TF) (Fig. 5 and Supplementary Data 5). CAGE analysis showed that the expression of FOXO3 in the MCF-7 time-course peaks at 30 min, and decreases below the basal level 60 min after induction (Supplementary Figure S3). FOXO3 is a known regulator of genes coordinating cell-cycle progression and apoptosis^43^, but the set of its direct targets is still largely unknown^44^. Our analysis suggests that FOXO3 influences the expression of the 23 promoters of Cluster 8, including DUSP5 and DUSP10. This result is supported by multiple ChIPSeq analyses on colon carcinoma cell lines, which identified a regulatory effect of FOXO3 on DUSP5^45^,^46^,^47^ and on DUSP10^44^. In addition, specific down–regulation of FOXO3 in the HRG-stimulated cells suggested that FOXO3 is repressed by some mechanism. It is known that an activity of FOXO3 is inhibited by Akt activation in a variety of cells^48^,^49^, and Akt is kept activated in the HRG-stimulated cells until 6 hrs (Fig. 1b), which suggests that a FOSL-dependent expression and Akt-dependent suppression of FOXO3 might shape the transient expression profiles of the genes belonging to Cluster 8. This result suggests a possible co-regulation of AP-1 complex and FOXO3, previously postulated in^47^.

*DUSP5* and *DUSP10* are not expressed in EGF-stimulated MCF-7 cell time-course (Fig. 2a), and DUSP5 protein levels are negligible, compared to HRG (Fig. 2b). Indeed the EGF regulatory network inferred by CIDER analysis completely lacks any regulatory interaction of *DUSP5* and *DUSP10*.

### Transcriptional regulation of FOS and FOSL1 in late HRG response

Cluster 9, a cluster of 21 promoters regulated by FOS and FOSL1 according to the CIDER analysis, has a delayed transcription that peaks between 120 and 180 min, and is sustained (slowly decreasing) up to 8 hours after induction (Fig. 7c). The pattern can be explained by assuming a positive contribution of FOSL1, still showing high levels of protein concentration in the late time points.

An interesting member of this cluster is the Retinoic Acid Receptor Alpha (RARA), whose expression and protein levels in EGF-induced time-course are significantly lower compared to HRG (Fig. 2a,b). Retinoic acid receptors (RARs) mediate retinoic acid effects, known to trigger antiproliferative effects in tumor cells, by directly regulating gene expression. Interestingly, RARA is known to antagonize the transcriptional regulation of the estrogen receptors for breast cancer-associated genes^50^,^51^. Based on motif enrichment analysis, Hua *et al.* showed that retinoic acid (RA) agonists decrease, while estrogen (E2) increases, the histone 3 acetylation for the AP-1 (FOS) regulatory element^50^. These studies indicate that the AP-1 mediates opposite effects of RAR and estrogen receptor for gene expression. Consistently, our results suggest that FOS and FOSL1 might cooperatively control the activity of RARA on its target gene expression at late time points; once activated, RARA may start negative control of estrogen receptor, whose transcription and protein phosphorylation levels (Ser118 activation) are consistently higher in the MCF-7 EGF-triggered time-courses at late time points, compared to HRG (Supplementary Figure S4).

The CIDER analysis found an additional TF, the tumor suppressor retinoid-induced interferon regulatory factor 1 (IRF1), enriched in Cluster 9 but not in Cluster 8 (Supplementary Data 5). *IRF1* expression peaks around 30 and 240 min after induction (Supplementary Figure S3), and might help sustaining the expression of Cluster 9. The regulation of RARA by IRF1 is of interest, since it is essential for the estrogen receptor and is critically required for TRAIL induction by RA^52^,^53^.

## Discussion

The CAGE expression time courses produced by the FANTOM5 consortium provide a rich description of the response of MCF-7 cells to HRG and EGF stimulation up to 8 hours after induction. Remarkably, after an initially qualitatively similar but quantitatively distinct MAPK pathway activation and similar SRF activity (up to 15 min.), the chain of regulation and the gene expression diverge significantly between the HRG and the EGF time courses, leading to different cellular phenotypes. In particular, after 30 min FOS is activated by HRG and not by EGF by prolonged ERK activation (Fig. 2b,c). This finding confirms the postulated all-or-none activation of FOS and the downstream regulatory cascade^7^. The high resolution of the FANTOM5 MCF-7 time courses (16 time points, 3 replicates) allowed us to profile by CIDER analysis the prominent regulatory patterns triggered by HRG and EGF ligands. Consistently with the respectively stronger and weaker elicitation of the MAPK pathway, the transcriptional regulation is stronger in the HRG time-course, as captured by the more complex structure of the CIDER regulatory network. The CIDER analysis revealed a prolonged regulatory effect, starting immediately after the induction, and extending to late time-points (up to 8 hours). Our results confirm that the SRF activation directly regulates the transcription of DUSP1 and DUSP2, and indirectly modulates the expression of DUSP5 and DUSP10 through the AP-1 complex (Fig. 5). This regulatory mechanism defines the negative feedback loop essential for the deactivation of the MAPK pathway through the dephosphorylation of the MAPK kinases (Fig. 1)^1^.

Our analysis also inferred the role of FOXO3 in the regulation of DUSP5 and DUSP10, in an interplay with the AP-1 complex. Moreover, according to CIDER the AP-1 complex, together with the IRF1 TF, promotes the expression of RARA, linking the ErbB pathway activation to the suppression of estrogen receptor activity, shown in the more aggressive phenotypes of breast cancer.

## Material and Methods

### MCF-7 cells

Preparation of MCF-7 cells for CAGE analysis was previously described^10^. Briefly, MCF-7 human breast cancer cell line was obtained from American Type Culture Collection (ATCC) and maintained in DMEM (Gibco BRL, Githersburg, MD) supplemented with 10% fetal bovine serum. Prior to growth hormone treatment, the cells were serum-starved for 16–24 hours, and then 10 nM EGF (PeproTech House, London, England) or 10 nM HRG-beta 176–246 (R&D Systems, Inc., Minneapolis, MN) were added and incubated for designated time period. We prepared the EGF or HRG-stimulated time-course samples at 0 (non-treated), 15 min, 30 min, 45 min, 60 min, 80 min, 100 min, 2 hr, 2.5 hr, 3 hr, 3.5 hr, 4 hr, 5 hr, 6 hr, 7 hr and 8 hr, to cover the early phase of cell differentiation and proliferation and to cover early, mid and delayed gene expression. All the cell samples were snap frozen in liquid nitrogen and provided for RNA extraction for CAGE analysis.

### CIDER analysis and in-silico and experimental validation of motif binding

CIDER infers the prominent patterns of regulatory interaction by motif-enrichment analysis over warped time-series clustering (Fig. 3). First, the distinct expression patterns are identified by unsupervised hierarchical clustering of time-series. Then, motif enrichment analysis is applied to each node in the clustering to identify the transcription factors likely to induce the transcription of the genes with similar expression patterns. The choice of parameters of the CIDER algorithm^11^ is detailed in Supplementary Material. For the definition of the flanking regions around the major TSS of each promoter, we used the stringent choice of [–300, +100] bp, consistently with the FANTOM5 time-course analysis^10^. The CIDER regulatory network is further refined by intersecting with the regulatory map derived by a less stringent flanking region interval of [–1500, +500] bp^54^.

The TF-gene associations within the SRF-rooted regulatory cascade inferred by CIDER and discussed in this study were confirmed *in-silico* by using the online LASAGNA tool^23^ with a P value significance threshold of *0.01*. We used ENCODE data^12^ to estimate an empirical FDR by counting the inferred SRF regulatory interactions supported by ChIPSeq experimental evidence. The dataset of raw signals and broad peaks of SRF binding in MCF-7 cells is available at the Gene Expression Omnibus repository, under the accession number GSM1010839.

### Protein abundance quantification by Western Blot

Protein abundance and phosphorylation levels were quantified by Western blot assay. Antibodies were obtained as follows; antibodies for phospho-EGFR (Y1068), phospho-ErbB2 (Y1221/1222), Akt, phospho-Akt (T308), ERK, phospho-ERK (T202/Y204), p38, phospho-p38 (T180/Y182), phospho-ESR (S167), JNK , phospho-JNK (T183/Y185), FOS, phospho-FOS (S32), phospho-JUN (S63), phospho-SRF, and SRF from Cell Signaling Technology; ESR and phospho-ESR (S118) from Upstate technology; EGFR from Fitzgerald, ErbB2 from Millipore; Antibodies for FRA1 (FOSl1), RARA, and DUSP5 from Santa Cruz Biotechnology. Time-course samples of EGF and HRG were run on different gels and then normalized by the relative ratio given from the blots on the same membrane (Supplementary Figures S1-S2). Two biological replicates were used for each time-point in each time-course. Protein bands were detected with chemi-luminescence and quantification was performed using Image Quant LAS 4000 (GE Healthcare).

## Additional Information

**How to cite this article**: Mina, M. *et al.* Promoter-level expression clustering identifies time development of transcriptional regulatory cascades initiated by ErbB receptors in breast cancer cells. *Sci. Rep.* **5**, 11999; doi: 10.1038/srep11999 (2015).

## Supplementary Material

Supplementary Information

Supplementary Data 1-6

Supplementary Data 7

## Figures and Tables

**Table 1:** Panel of regulatory interactions inferred by CIDER for the MCF-7 HRG and EGF time-courses.

**Regulatory interaction**	**Novel in MCF-7**	**Validated in**	
		**MCF-7**	**other tissues**
SRF -> FOS		2	16–22
SRF -> DUSP1	x		16
SRF -> DUSP2	x		16
SRF -> EGR1		55	16–22
SRF -> EGR2	x		16–22
SRF -> EGR4	x		16
SRF -> NR4A1	x		16
FOS -> FOSL1		36, 37	38, 39
AP1 -> DUSP5		36	
AP1 -> DUSP10	x		
AP1 -> RARA		56	
FOXO3 -> DUSP5	x		45–47
FOXO3 -> DUSP10	x		44
IRF1 -> RARA	x		

The interactions in bold are present in both the MCF-7 EGF and HRG time-courses. Each interaction is annotated with the references to the literature experimentally supporting the existence of the interaction in the MCF-7 time-course and/or in other tissues.

## References

[b1] Avraham R. & Yarden Y. Feedback regulation of EGFR signalling: decision making by early and delayed loops. Nat. Rev. Mol. Cell Bio. 12, 104–17 (2011). 21252999 10.1038/nrm3048

[b2] Duan R., Xie W., Li X., McDougal A. & Safe S. Estrogen regulation of c-fos gene expression through phosphatidylinositol-3-kinase-dependent activation of serum response factor in MCF-7 breast cancer cells. Biochem. Bioph. Res. Co. 294, 384–94 (2002). 10.1016/S0006-291X(02)00499-0 12051724

[b3] Marshall C. J. Specificity of receptor tyrosine kinase signaling: transient versus sustained extracellular signal-regulated kinase activation. Cell 2, 179–185 (1995). 10.1016/0092-8674(95)90401-8 7834738

[b4] Yarden Y. & Sliwkowski M. Untangling the ErbB signalling network. Nat Rev. Mol. Cell. Bio. 2, 127–137 (2001). 11252954 10.1038/35052073

[b5] Saeki Y. *et al.* Ligand-specific sequential regulation of transcription factors for differentiation of MCF-7 cells. BMC Genomics 10, 545 (2009). 19925682 10.1186/1471-2164-10-545 PMC2785842

[b6] Nagashima T. *et al.* Quantitative transcriptional control of ErbB receptor signaling undergoes graded to biphasic response for cell differentiation. J. Biol. Chem. 282, 4045–56 (2007). 17142811 10.1074/jbc.M608653200

[b7] Nakakuki T. *et al.* Ligand-specific c-Fos expression emerges from the spatiotemporal control of ErbB network dynamics. Cell 141, 884–896 (2010). 20493519 10.1016/j.cell.2010.03.054 PMC2888034

[b8] Murphy L. O. & Blenis J. MAPK signal specificity: the right place at the right time. Trends Biochem. Sci. 31, 268–75 (2006). 16603362 10.1016/j.tibs.2006.03.009

[b9] Posern G. & Treisman R. Actin’ together: serum response factor, its cofactors and the link to signal transduction. Trends. Cell Biol. 16, 588–96 (2006). 17035020 10.1016/j.tcb.2006.09.008

[b10] Arner E. *et al.* Transcribed enhancers lead waves of coordinated transcription in transitioning mammalian cells. Science 347, 1010–1014 (2015). 25678556 10.1126/science.1259418 PMC4681433

[b11] Mina M., Jurman G. & Furlanello C. CIDER: a pipeline for detecting waves of coordinated transcriptional regulation in gene expression time-course data. bioRxiv (2015).

[b12] ENCODE Project Consortium. An integrated encyclopedia of DNA elements in the human genome. Nature 489, 57–74 (2012). 22955616 10.1038/nature11247 PMC3439153

[b13] Murphy L. O., MacKeigan J. P. & Blenis J. A network of immediate early gene products propagates subtle differences in mitogen-activated protein kinase signal amplitude and duration. Mol. Cell Biol. 24, 144–53 (2004). 14673150 10.1128/MCB.24.1.144-153.2004 PMC303364

[b14] Caunt J. C. & Keyse S. M. Dual-specificity MAP kinase phosphatases (MKPs). Shaping the outcome of MAP kinase signaling. FEBS J. 280, 489–504 (2013). 22812510 10.1111/j.1742-4658.2012.08716.x PMC3594966

[b15] Zipfel P. F., Decker E. L., Holst C. & Skerka C. The human zinc finger protein EGR-4 acts as autoregulatory transcriptional repressor. Biochim. Biophys. Acta 1354, 134–144 (1997). 9396630 10.1016/s0167-4781(97)00084-5

[b16] Iacono G., Altafini C. & Torre V. Early phase of plasticity-related gene regulation and SRF dependent transcription in the hippocampus. PloS One 8, e68078 (2013). 23935853 10.1371/journal.pone.0068078 PMC3720722

[b17] Kim K. H., Min Y. K., Baik J. H., Lau L. F., Chaqour B. & Chung K. C. Expression of angiogenic factor Cyr61 during neuronal cell death via the activation of c-Jun N-terminal Kinase and Serum Response Factor. J. Biol. Chem. 278, 13847–13854 (2003). 12576482 10.1074/jbc.M210128200

[b18] Lindecke A. *et al.* Long-term depression activates transcription of immediate early transcription factor genes: involvement of serum response factor/Elk-1. Eur. J. Neurosci. 24, 555–563 (2006). 16903857 10.1111/j.1460-9568.2006.04909.x

[b19] Benito E., Valor L. M., Jimenez-Minchan M., Huber W. & Barco A. cAMP response element-binding protein is a primary hub of activity-driven neuronal gene expression. J. Neurosci. 31, 18237–18250 (2011). 22171029 10.1523/JNEUROSCI.4554-11.2011 PMC6623905

[b20] Tullai J. W., Schaffer M. E., Mullenbrock S., Kasif S. & Cooper G. M. Identification of transcription factor binding sites upstream of human genes regulated by the phosphatidylinositol 3-kinase and MEK/ERK signaling pathways. J. Biol. Chem. 279, 20167–20177 (2004). 14769801 10.1074/jbc.M309260200

[b21] O’Sullivan N. C., Pickering M., Di Giacomo D., Loscher J. S. & Murphy K. J. Mkl transcription cofactors regulate structural plasticity in hippocampal neurons. Cereb. Cortex 20, 1915–1925 (2010). 20016002 10.1093/cercor/bhp262

[b22] Kumar V., Fahey P. G., Jong Y. J., Ramanan N. & O’Malley K. L. Activation of the intracellular metabotropic glutamate receptor 5 in striatal neurons leads to upregulation of genes associated with sustained synaptic transmission including Arc/Arg3.1. J. Biol. Chem. 287, 5412–5425 (2011). 22179607 10.1074/jbc.M111.301366 PMC3285320

[b23] Lee C. & Huang C. LASAGNA-Search: an integrated web tool for transcription factor binding site search and visualization. Biotechniques 54, 141–153 (2013). 23599922 10.2144/000113999

[b24] The FANTOM Consortium and the Riken Omics Science Center. The transcriptional network that controls growth arrest and differentiation in a human myeloid leukemia cell line. Nat. Genet. 41, 553–62 (2009). 19377474 10.1038/ng.375 PMC6711855

[b25] Prywes R., Dutta A., Cromlish J. A. & Roeder R. G. Phosphorylation of serum response factor, a factor that binds to the serum response element of the c-FOS enhancer. P. Nat. Acad. Sci. USA 85, 7206–7210 (1988). 10.1073/pnas.85.19.7206 PMC282153 2845402

[b26] Cohen D. R. & Curran T. FRA-1: a serum-inducible, cellular immediate-early gene that encodes a fos-related antigen. Mol. Cell Biol. 8, 2063–2069 (1988). 3133553 10.1128/mcb.8.5.2063-2069.1988 PMC363385

[b27] Foletta V. C., Sonobe M. H., Suzuki T., Endo T., Iba H. & Cohen D. R. Cloning and characterisation of the mouse fra-2 gene. Oncogene 9, 3305–3311 (1994). 7936655

[b28] Matsui M., Tokuhara M., Konuma Y., Nomura N. & Ishizaki R. Isolation of human fos-related genes and their expression during monocyte-macrophage differentiation. Oncogene 5, 249–55 (1990). 2107490

[b29] Nishina H., Sato H., Suzuki T., Sato M. & Iba H. Isolation and characterization of fra-2, an additional member of the fos gene family. P. Nat. Acad. Sci. USA 87, 3619–3623 (1990). 10.1073/pnas.87.9.3619 PMC53953 2110368

[b30] Zerial M., Toschi L., Ryseck R. P., Schuermann M., Müller R. & Bravo R. The product of a novel growth factor activated gene, fos B, interacts with JUN proteins enhancing their DNA binding activity. EMBO J. 8, 805–813 (1989). 2498083 10.1002/j.1460-2075.1989.tb03441.x PMC400877

[b31] Hirai S. I., Ryseck R. P., Mechta F., Bravo R. & Yaniv M. Characterization of junD: a new member of the jun proto-oncogene family. EMBO J. 8, 1433–1439 (1989). 2504580 10.1002/j.1460-2075.1989.tb03525.x PMC400971

[b32] Nishimura T. & Vogt P. K. The avian cellular homolog of the oncogene jun. Oncogene 3, 659–663 (1988). 2577867

[b33] Ryder K., Lanahan A., Perez-Albuerne E. & Nathans D. jun-D: a third member of the jun gene family. P. Nat. Acad. Sci. USA 86, 1500–1503 (1989). 10.1073/pnas.86.5.1500 PMC286724 2493644

[b34] Karin M., Liu Z. G. & Zandi E. AP-1 function and regulation. Curr. Opin. Cell Biol. 9, 240–6 (1997). 9069263 10.1016/s0955-0674(97)80068-3

[b35] Farioli-Vecchioli S. *et al.* Impaired terminal differentiation of hippocampal granule neurons and defective contextual memory in PC3/Tis21 knockout mice. PLoS One 4, e8339 (2009). 20020054 10.1371/journal.pone.0008339 PMC2791842

[b36] Nunes-Xavier C. E. *et al.* Differential up-regulation of MAP kinase phosphatases MKP3/DUSP6 and DUSP5 by Ets2 and c-Jun converge in the control of the growth arrest versus proliferation response of MCF-7 breast cancer cells to phorbol ester. J. Biol. Chem. 285, 26417–30 (2010). 20554528 10.1074/jbc.M110.121830 PMC2924073

[b37] Verde P., Casalino L., Talotta F., Yaniv M. & Weitzman J. B. Deciphering AP-1 function in tumorigenesis: fra-ternizing on target promoters. Cell Cycle 6, 2633–9 (2007). 17957143 10.4161/cc.6.21.4850

[b38] Young M. R. & Colburn N. H. Fra-1 a target for cancer prevention or intervention. Gene 379, 1–11 (2006). 16784822 10.1016/j.gene.2006.05.001

[b39] Bergers G., Graninger P., Braselmann S., Wrighton C. & Busslinger M. Transcriptional activation of the fra-1 gene by AP-1 is mediated by regulatory sequences in the first intron. Mol. Cell Biol. 15, 3748–3758 (1995). 7791782 10.1128/mcb.15.7.3748 PMC230613

[b40] Pulido R. & Hooft van Huijsduijnen R. Protein tyrosine phosphatases: dual-specificity phosphatases in health and disease. FEBS J. 275, 848–866 (2008). 18298792 10.1111/j.1742-4658.2008.06250.x

[b41] Patterson K. I., Brummer T., O’Brien P. M. & Daly R. J. Dual-specificity phosphatases: critical regulators with diverse cellular targets. Biochem. J. 418, 475–489 (2009). 19228121 10.1042/bj20082234

[b42] Miller D. V. *et al.* Utilizing Nottingham Prognostic Index in microarray gene expression profiling of breast carcinomas. Modern Pathol. 17, 756–764 (2004). 10.1038/modpathol.3800114 15073601

[b43] Salih D. A. & Brunet A. FoxO transcription factors in the maintenance of cellular homeostasis during aging. Curr. Opin. Cell Biol. 20, 126–36 (2008). 18394876 10.1016/j.ceb.2008.02.005 PMC2387118

[b44] Webb A. E. *et al.* FOXO3 shares common targets with ASCL1 genome-wide and inhibits ASCL1-dependent neurogenesis. Cell Reports 4, 477–91 (2013). 23891001 10.1016/j.celrep.2013.06.035 PMC3838667

[b45] Delpuech O. *et al.* Induction of Mxi1-SR alpha by FOXO3a contributes to repression of Myc-dependent gene expression. Mol. Cell Biol. 27, 4917–30 (2007). 17452451 10.1128/MCB.01789-06 PMC1951505

[b46] Eijkelenboom A., Mokry M., Smits L. M., Nieuwenhuis E. E. & Burgering B. M. T. FOXO3 selectively amplifies enhancer activity to establish target gene regulation. Cell Reports 5, 1664–78 (2013). 24360957 10.1016/j.celrep.2013.11.031

[b47] Eijkelenboom A. *et al.* Genome-wide analysis of FOXO3 mediated transcription regulation through RNA polymerase II profiling. Mol. Syst. Biol. 9, 638 (2013). 23340844 10.1038/msb.2012.74 PMC3564262

[b48] Brunet A. *et al.* Akt promotes cell survival by phosphorylating and inhibiting a Forkhead transcription factor. Cell 96, 857–68 (1999). 10102273 10.1016/s0092-8674(00)80595-4

[b49] Carter M. E. & Brunet A. FOXO transcription factors. Curr. Biol. 17, R113–4 (2007). 17307039 10.1016/j.cub.2007.01.008

[b50] Hua S., Kittler R. & White K. P. Genomic antagonism between retinoic acid and estrogen signaling in breast cancer. *Cell* 137, 1259–71 (2009). 10.1016/j.cell.2009.04.043 PMC3374131 19563758

[b51] Ross-Innes C. S. *et al.* Cooperative interaction between retinoic acid receptor-alpha and estrogen receptor in breast cancer. Gene Dev. 24, 171–82 (2010). 20080953 10.1101/gad.552910 PMC2807352

[b52] Clarke N., Jimenez-Lara A. M., Voltz E. & Gronemeyer H. Tumor suppressor IRF-1 mediates retinoid and interferon anticancer signaling to death ligand TRAIL. EMBO J. 23, 3051–60 (2004). 15241475 10.1038/sj.emboj.7600302 PMC514919

[b53] Alvarez S., Germain P., Alvarez R., Rodríguez-Barrios F., Gronemeyer H. & de Lera A. R. Structure, function and modulation of retinoic acid receptor beta, a tumor suppressor. Int. J. Biochem. Cell B. 39, 1406–15 (2007). 10.1016/j.biocel.2007.02.010 17433757

[b54] Veerla S., Ringnér M. & Höglund M. Genome-wide transcription factor binding site/promoter databases for the analysis of gene sets and co-occurrence of transcription factor binding motifs. BMC Genomics 11, 145 (2010). 20193056 10.1186/1471-2164-11-145 PMC2841680

[b55] Chen C. C., Lee W. R. & Safe S. Egr-1 is activated by 17beta-estradiol in MCF-7 cells by mitogen-activated protein kinase-dependent phosphorylation of ELK-1. J. Cell. Biochem. 93, 1063–74 (2004). 15449318 10.1002/jcb.20257

[b56] Yang L., Kim H., Munoz-Medellin D., Reddy P. & Brown P. H. Induction of retinoid resistance in breast cancer cells by overexpression of cJun. Cancer Res. 57, 4652–61 (1997). 9377582

