# Supplementary material for: Promoter-level expression clustering identifies time development of transcriptional regulatory cascades initiated by ErbB receptors in breast cancer cells
Source: Sci Rep. 2015 Jul 16;5:11999. doi: 10.1038/srep11999 (PMC4503981; doi:10.1038/srep11999)
Supplement: Supplementary Information [file srep11999-s1.pdf]

Supplementary Material

Promoter-level expression clustering identifies time development of transcriptional regulatory cascades initiated by ErbB receptors in breast cancer cells

Marco Mina[1,#], Shigeyuki Magi[2,#], Giuseppe Jurman[1], Masayoshi Itoh[3,4,5], Hideya Kawaji[3,4,5], Timo Lassmann[3,4,6], Erik Arner[3,4,8], Alistair R.R. Forrest[3,4], Piero Carninci[3,4], Yoshihide Hayashizaki[3,5], Carsten O Daub[3,4,7], the FANTOM Consortium, Mariko Okada-Hatakeyama[2,*], and Cesare Furlanello[1,*]

[1] Fondazione Bruno Kessler, Via Sommarive 18, I-38123 Povo, Trento, Italy
[2] Laboratory for Integrated Cellular Systems, RIKEN Center for Integrative Medical Sciences (IMS), Tsurumi-ku, Yokohama, Kanagawa 230-0045, Japan
[3] RIKEN Center for Life Science Technologies (Division of Genomic Technologies) (CLST (DGT)), 1-7-22 Suehiro-cho, Tsurumi-ku, Yokohama, Kanagawa 230-0045, Japan
[4] RIKEN Omics Science Center (OSC), 1-7-22 Suehiro-cho, Tsurumi-ku, Yokohama 230-0045, Japan
[5] RIKEN Preventive Medicine and Diagnosis Innovation Program (PMI), 2-1 Hirosawa, Wako-shi, Saitama 351-0198, Japan
[6] Telethon Kids Institute, The University of Western Australia, 100 Roberts Road, Subiaco, WA 6008, Australia
[7] Department of Biosciences and Nutrition and Science for Life Laboratory, Karolinska Institutet, Stockholm, SE-141 86, Sweden
[8] Department of Medicine at Karolinska Institutet and Center for Metabolism and Endocrinology at Karolinska University Hospital, 141 86, Stockholm, Sweden

[#] These authors contributed equally to this work.

* To whom correspondence should be addressed. Tel: +39 0461314580; Fax: +39 0461314591; Email: furlan@fbk.eu. Correspondence may also be addressed to Mariko Okada-Hatakeyama. Tel: +81 455039302; Fax: +81 455039613 ; Email: marikoh@rcai.riken.jp.

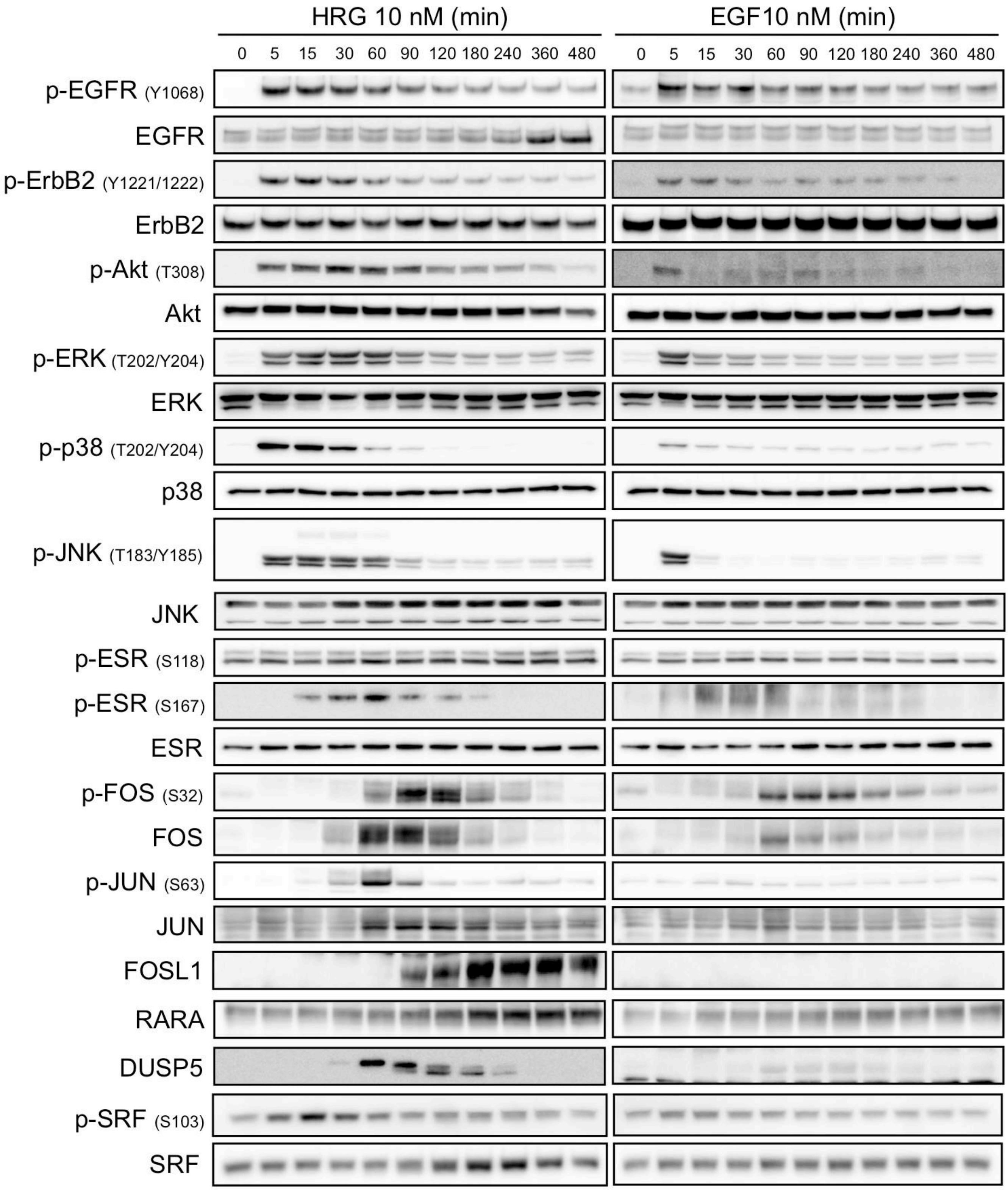

Supplementary Figure S1. Time-course Western blots on EGF and HRG on different membranes.

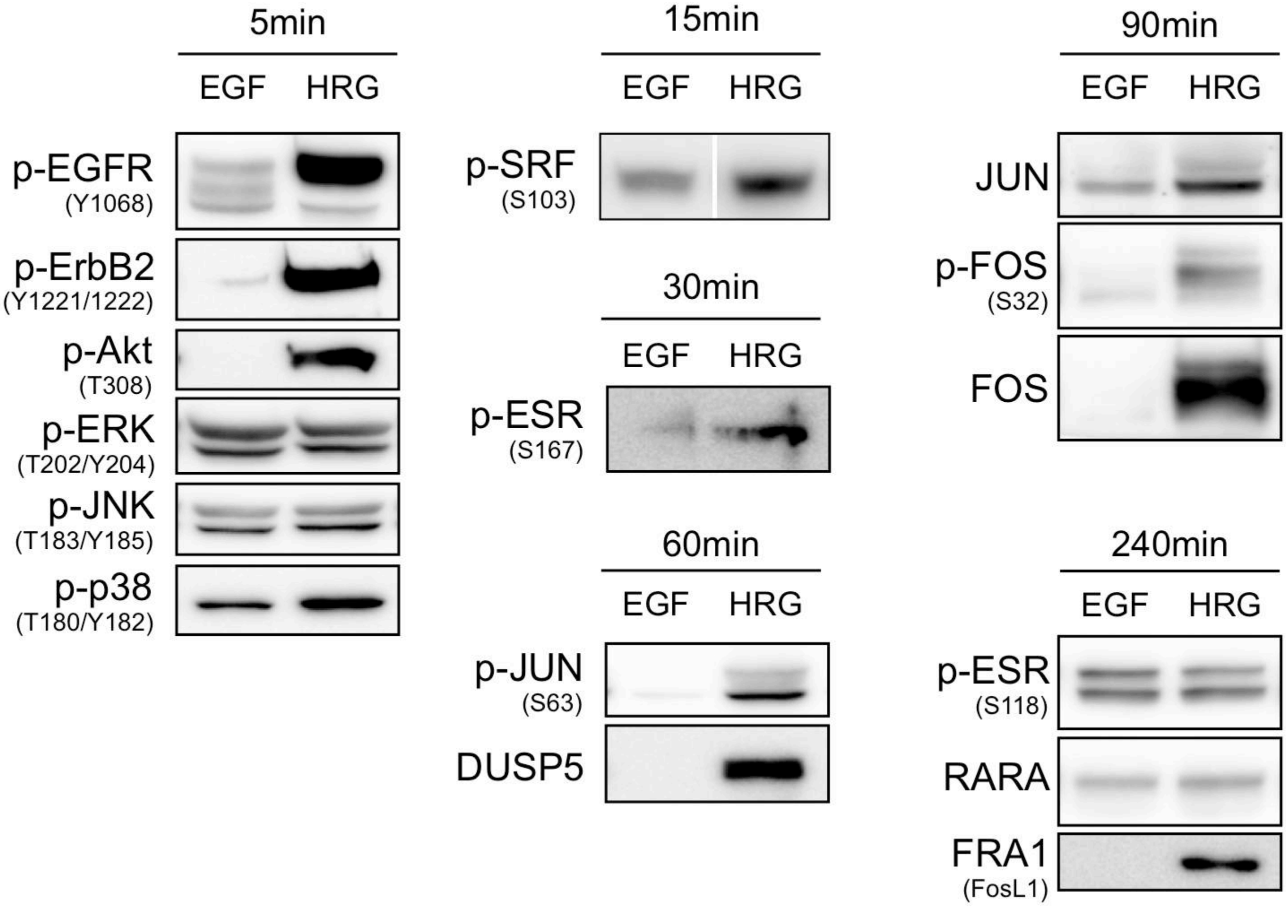

Supplementary Figure S2. The ratio of band intensities for EGF vs HRG was used for normalization of time-course Western blots on different membranes.

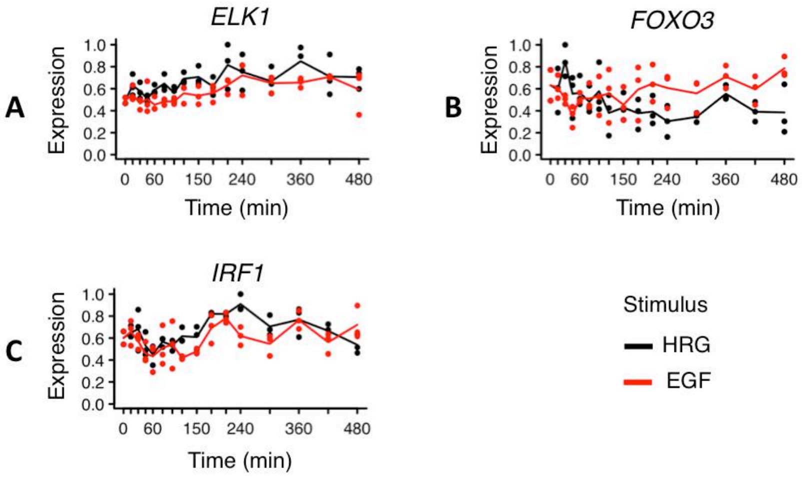

Supplementary Figure S3. (A) CAGE expression of *ELK1*, IEG activated by MAPK pathway together with SRF. ELK1 expression is similar between EGF and HRG time courses. (B) CAGE expression of the *FOXO3* TF, enriched in cluster 8. After an initial peak at 30 min after induction, *FOXO3* expression decreases in HRG time-course, with an opposite trend respect to EGF induction. (C) CAGE expression of the *IRF1* TF, enriched in cluster 9. *IRF1* expression peaks at 30 and 240 min after HRG induction, and is similar between EGF and HRG time courses. Each dot represents the quantified values in an independent experiment, and lines represent the average of these values.

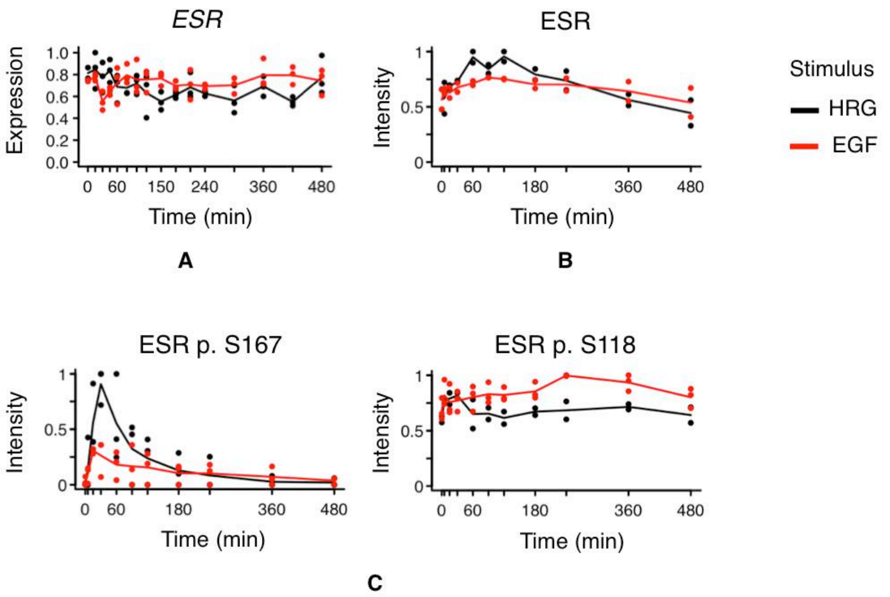

Supplementary Figure S4. (A) CAGE expression of estrogen receptor (ESR) induced by EGF (red)- and HRG (black)-stimulated MCF-7 cells. (B) Western blot analysis of ESR performed to quantify protein levels after the stimulation of the same ligands (10 nM). (C) Western blot analysis of ESR performed to quantify protein phosphorylation levels after the stimulation of the same ligands (10 nM). Each dot represents the quantified values in an independent experiment, and lines represent the average of these values.

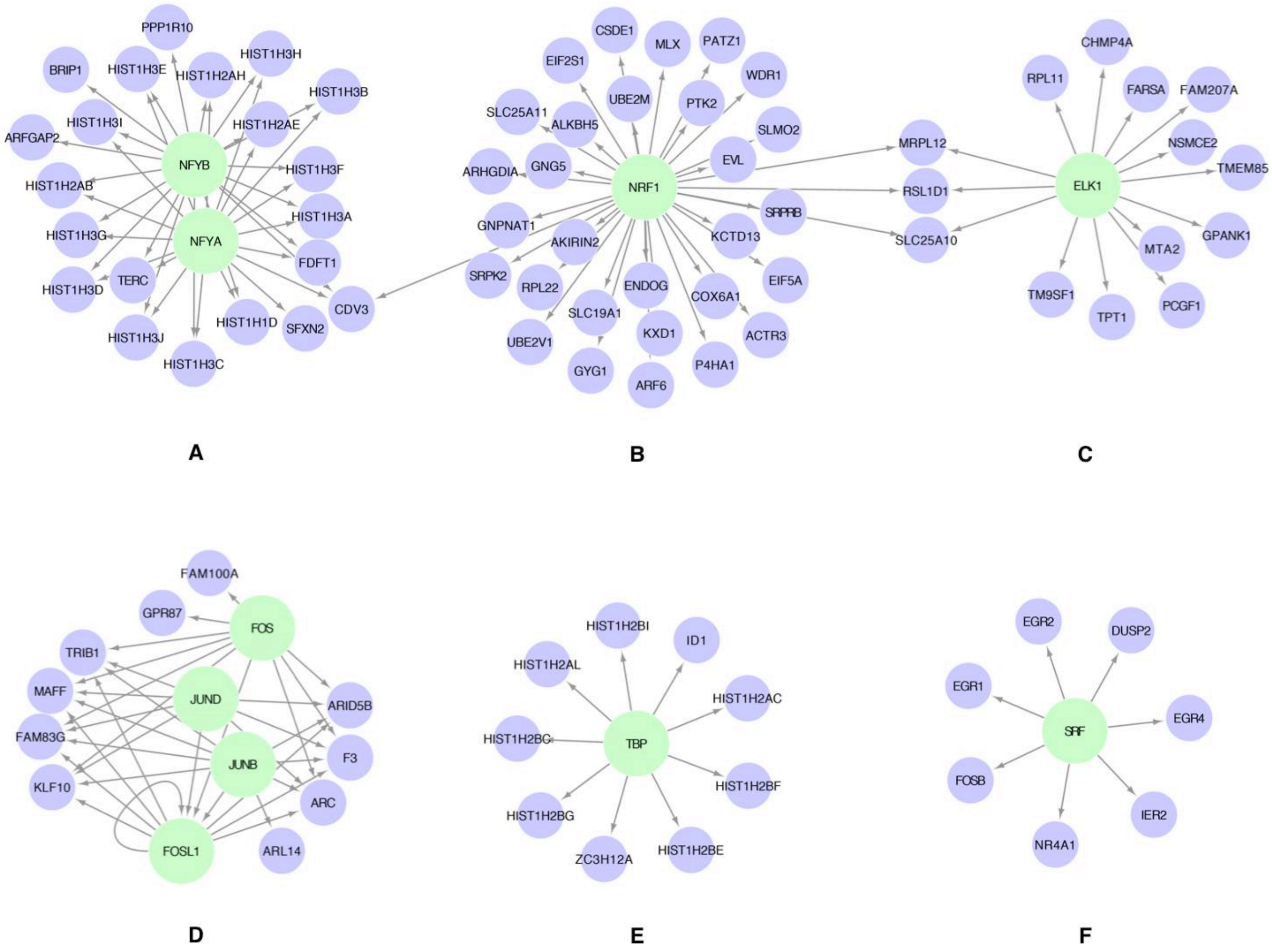

Supplementary Figure S5. Transcriptional regulatory modules consistently found by both MARA and CIDER analysis on the MCF-7 HRG time-course data. Light green nodes represent the TFs regulating the blue downstream genes (in blue). (**A**) NFYA/B regulon. (**B**) NRF1 regulon. (**C**) ELK1 regulon. (**D**) FOS-FOSL1-JUNB/D regulon. (**E**) TBP regulon. (**F**) SRF regulon.

A. Early response pattern

Cluster 3: 36 promoters

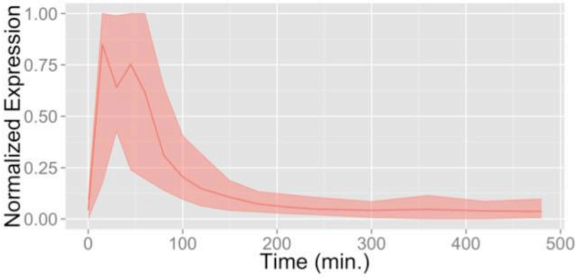

FOS (2353)
IER2 (9592)

B. Intermediate response pattern

Cluster 4: 205 promoters; Cluster 5: 65 promoters

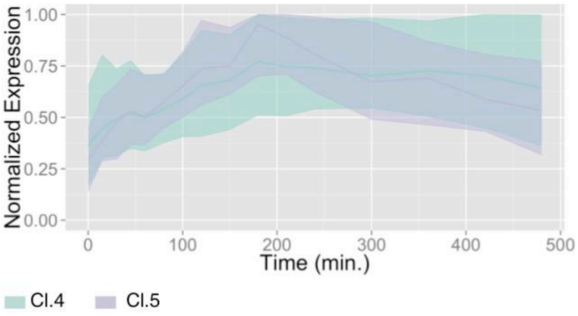

GO Term Id	GO Term Description	Corrected P.value	
		Cl. 4	Cl. 5
0012501	Programmed cell death	**3.3 e-06**	**9.3 e-05**
0071363	Response to growth factor	**6.1 e-05**	**6.6 e-04**
0001568	Blood vessel development	> 0.01	**4.7 e-04**
0001525	Angiogenesis	> 0.01	**2.8 e-05**

ITGB6 (3694)
ABCC1 (4363)
ACVR1 (90)
BCL10 (8915)
BLNK (29760)
CTSL2 (1515)

DOK7 (285489)
DOLK (22845)
DOT1L (84444)
RGAG4 (340526)
TGFB2 (7042)
TNFRSF1A (7132)

HIPK2 (28996)
KPNA3 (3839)
LAMC1 (3915)
MAPK7 (5598)
NFXL1 (152518)
NUAK2 (81788)

PLEKHG2 (64857)
PPP1CB (5500)
RELT (84957)
ENC1 (8507)
GATA6 (2627)
GEMIN4 (50628)

C. Late response pattern

Cluster 6: 20 promoters

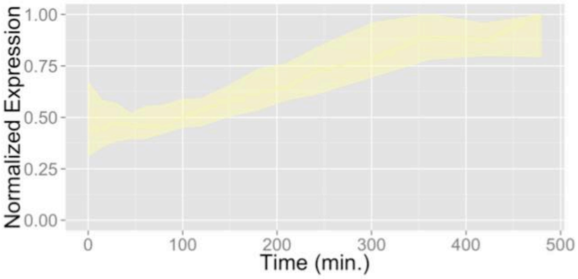

GO Term Id	GO Term Description	Corr. P.value
0040011	Locomotion	**4.4 e-04**
0042060	Wound healing	**5.0 e-04**
0006928	Cellular component movement	**1.5 e-03**

SLC25A11 (8402)
ACTB (60)
ADAP1 (11033)
ATP1B1 (481)
CCDC86 (79080)
CD276 (80381)

CNN2 (1265)
LIMK1 (3984)
SLC2A4RG (56731)
NEK6 (10783)
PDGFA (5154)
PTP4A1 (7803)

SLC27A4 (10999)
SLC7A5 (8140)
SULT2B1 (6820)
TGFB1 (7040)
MLX (6945)
SLC19A1 (6573)

Supplementary Figure S6. The expression patterns of the four clusters enriched for Jaspar MA0476.1 motif (SRF transcription factor) in the HRG time-course. Clusters 4-6 were only found enriched using the less restrictive flanking region parameters [-1500, +500]bp. Left: representation of the average expression pattern (lines) and 10%-90% quantile interval (colored regions). Right: selection of Gene Ontology terms most enriched (top tables) and of genes (bottom list of gene names and Entrez ids) for each pattern. A. Early response pattern. B. Intermediate response pattern. C. Late response pattern. No Gene Ontology enrichment analysis was performed for Cluster 3 as it only contains two genes.

**Regulatory Interaction**	**SRF Peak in ChiPSeq data**	**HRG time-course**	**EGF time-course**
**SRF -> IER2**	**X**	**X (cl 3)**	**X**
**SRF -> DUSP2**	**X**	**X (cl 1)**	**X (cl 2)**
**SRF -> EGR1**	**X**	**X (cl 1)**	**X (cl 2)**
**SRF -> EGR2**	**X**	**X (cl 1)**	**X (cl 2)**
**SRF -> EGR4**	**X**	**X (cl 1)**	**X (cl 2)**
**SRF -> FOS**	**X**	**X (cl 1)**	**X (cl 2)**
**SRF -> FOSB**	**X**	**X (cl 1)**	**X (cl 2)**
**SRF -> NR4A1**	**X**	**X (cl 1)**	**X (cl 2)**
**SRF -> DUSP1**		**X (cl 1)**	**X (cl 2)**
**SRF -> CTGF**		**X (cl 1)**	**X (cl 2)**
SRF -> LDLR	X		X (cl 2)
SRF -> MIR22HG	X		X (cl 2)
SRF -> MYC	X		X (cl 2)
SRF -> SCARNA2			X (cl 2)
SRF -> SLC30A1			X (cl 2)
SRF -> KLF2			X (cl 2)
SRF -> SIK1			X (cl 2)
SRF -> CSRNP1			X (cl 2)
SRF -> NCOA7	X		X
SRF -> MNT	X		X
SRF -> INPP5K	X		X
SRF -> PTGER4	X		X
SRF -> TLE3	X		X
SRF -> HIST1H2BO			X
Total correct	16 on 24 (67 %)	8 on 10 (80 %)	16 on 24 (67 %)

Supplementary Table S1. Validation of the SRF regulatory interactions inferred by CIDER for either the MCF-7 HRG or the EGF time-course. The interactions inferred for both time-courses are in bold. Each interaction is annotated for the presence of a SRF binding peak in the ENCODE ChIPSeq dataset GSM1010839. The third and fourth columns are annotated with the proper cluster id.

	# targets CIDER	# targets MARA	Overlap	Fisher's test Two-tails P value	Barnard's test Two-tails P value
**SRF (all CIDER targets)**	10	56	7	$8.42e^{-12}$	0.0013
**SRF (only Cluster 1)**	9	56	6	$4.70e^{-10}$	0.0012
**ELK1**	331	792	14	$< 2.2e^{-16}$	$2.29e^{-08}$
**FOS-FOSL1-JUNB/D (union of targets)**	74	76	11	$7.86e^{-08}$	$5.06e^{-05}$
**NFYA - NFYB**	405	330	22	0.029	0.056
**TBP**	122	98	9	0.003	0.023
**NRF1**	1008	573	33	$< 2.2e^{-16}$	$4.58e^{-26}$

Supplementary Table S2. Statistics of the six overlapping modules between MARA and CIDER TRNs.

	**CIDER**	**MARA**
**Input**	Gene expression time-course and known TF binding motifs	
**Output**	1. Prominent expression patterns<br>2. TRN of the master regulator TFs of each expression pattern	1. TF regulatory activity profiles<br>2. Weighted TRN of TF-gene regulatory interactions
**Methodology**	1. Time series clustering<br>2. Motif enrichment analysis<br>3. Combination of the TF regulons in transcriptional cascades	1. Estimation of the TF regulatory activity by fitting a multivariate linear model<br>2. Estimation of TF-gene pairs regulation based on expression correlation

Supplementary Table S3. Comparison of CIDER and MARA methodologies.

1. Description of the CIDER analysis pipeline

CIDER [1] consists of two steps: first, the distinct expression patterns are identified by unsupervised clustering of time series. Then, motif enrichment analysis is performed on the clusters, to identify the transcription factors likely to induce the transcription of the genes with similar expression pattern (Figure 3). In details:

1. Time series clustering: A hierarchical clustering approach is adopted to identify expression patterns from longitudinal molecular data. Paragraphs *1.A-1.C* provide details on preprocessing, distance, and specific hierarchical clustering methods adopted in CIDER.

1.A Time series preprocessing: time series are constrained between 0 and 1 by dividing each time series by its maximum value, in order to apply distances that require time series normalization (see step 1.B). Each replicate is normalized separately, to avoid penalizing replicates with lower tag per million (tpm) counts, and focus on shape patterns instead of magnitude levels. To reduce the impact of outliers in short time series, instead of averaging the replicates, they are concatenated obtaining longer time series (47 time-points for the MCF-7 time-courses).

1.B Time series distance: to overcome known issues with correlation-based methods [2,3], as an appropriate distance for time series clustering, CIDER adopts the Euclidean-based Dynamic Time Warping (DTW) distance [4], The application of DTW on transcriptomics time series profiling has been already demonstrated on microarray platforms [5]. DTW alignment is constrained to align time-points within each replicate, with a Sakoe-Chiba band of width 1 as global constraint [6]. As a further refinement, we implemented in CIDER the Complexity Invariant (CID) correction for DTW (CIDDTW method), one of the latest installations of DTW, proposed as a generalization that accounts for signal complexity [7]. In details, the CIDDTW method introduces a correction term penalizing signals with low variability; the rationale is that for standard distance measures (Euclidean, correlation, non-corrected DTW) pairs of complex objects tend to be gauged further apart than pairs of simple objects, introducing errors in classification. The R package *dtw* [8] was extended to implement the CIDDTW, and the R package *parallel* used to parallelize the task on multiple processors.

1.C Hierarchical clustering: Time series are clustered in CIDER by means of the complete-linkage agglomerative hierarchical clustering algorithm. The R package *fastcluster* [9] is used to perform hierarchical clustering on thousands of time series, instead of the default R *hclust* hierarchical clustering solution.

2. Multilevel Motif Enrichment analysis: CIDER adopts motif enrichment as the pruning rule for clustering. In general, motif enrichment is defined a class of techniques to identify motifs with a significant number of binding sites in a set of DNA sequences (i.e. in the flanking regions of the transcription start sites of a group of genes). Here we detail the specific enrichment technique and parameters used in the pruning phase for the MCF-7 analysis.

2.A Motif Enrichment: The hierarchical clustering structure produced at step 1.C is used as starting point to perform a multi-level motif enrichment analysis. Each node in the cluster is tested for enriched motifs using the AME tool, part of the MEME software suite [10] with default parameters. The AME application is parallelized by using the GNU *parallel* utility [11].

For the MCF-7 analysis, a total of 205 motif consensus matrices were downloaded from the Jaspar website [12] on November 2013, and processed according to the instructions provided at the MEME website to be compatible with AME input formats (Supplementary Data 7). The flanking regions around the major Transcription Start Site (TSS) of each promoter were extracted from the Human Genome h.19 using SAMtools [13]. In particular, in this work we considered both the stringent choice of [-300, +100] bp, consistently with the FANTOM5 time-course analysis [14], and of [-1500, +500] bp [15]. Overlapping flanking regions were merged in a single sequence to avoid overcounting binding sites. For each cluster, a 10-fold set of random sequences selected from the pool of flanking regions of all the promoters identified in the FANTOM5 dataset was used as background set for motif enrichment. The GC content of query and background sets were matched to avoid the GC content bias in motif enrichment, as described in [16]. For each motif, AME counts the number of binding sites in the foreground and background sets of flanking regions, and it evaluates the over-representation in the foreground set by Fisher's exact test.

2.B Enrichment-based pruning: The enrichment associations computed by AME on the MCF-7 data were pruned to retain only the most relevant associations between motif activity and expression patterns, by applying the criterion typically used in AME and other reference motif enrichment tools [17-19]. The association between a cluster and a motif was deemed significant if the Fisher test P value < *0.01* (absolute thresholding) [17,18], and greater than *1.5* standard deviations the mean of the enrichment P values of the other transcriptional motifs (relative thresholding) [19].

2. Comparison of CIDER and MARA results

We apply the CIDER analysis to the MCF-7 HRG time-course, which belongs to the FANTOM5 time-course dataset collection. The same time-course has been evaluated by Motif Activity Response Analysis (MARA) [20], with results described in the FANTOM5 time-course main paper [14]. Here we provide a synthetic comparison between the two methods,

showing that the main transcriptional modules identified by CIDER for the MCF-7 HRG time-course are also supported by MARA.

In general, both methods are designed to infer the transcription factor activity from gene expression time series and TF binding motif data. CIDER aims at identifying the most prominent expression patterns and infer the TFs responsible for the coordinated gene expression. MARA, instead, has been developed to estimate the regulatory activity of each TF and its effect on the single genes. A comparison of the two algorithms is presented in Supplementary Table S3.

Despite different background methodologies and purposes, CIDER and MARA analyses of the MCF-7 HRG time-course generated two transcriptional regulatory networks (TRNs) whose overlap is not randomly distributed on sparse interactions, but is organized in 8 regulatory modules controlled by 12 TFs. In particular, three of the overlapping modules are regulated by SRF, ELK1 and AP-1 complex involved in the regulatory cascade triggered by the MAPK activation (Figures 1 and 5).

In details, the MCF-7 HRG transcriptional regulatory network produced by CIDER counts 8470 regulatory interactions between 2575 promoters (1409 genes). The MARA network, instead, is composed by 8861 interactions between 3005 genes. The 136 interactions in overlap between the two regulatory networks are organized in 6 regulatory modules (Supplementary Figure S5). In particular, the overlapping modules regulated by the AP-1 complex (Figure S5D) and SRF (Figure S5F) have a statistically significant overlap between MARA and CIDER (Supplementary Table S2).

3. Additional clusters enriched for SRF in the HRG time-course

A second cluster of 36 promoters (Cluster 3), characterized by an immediate expression pattern, was found enriched for SRF by CIDER in the HRG time-course (Supplementary Figure S6A). The expression of Cluster 3 is consistent with the early peak phosphorylation dynamics of SRF (Figure 2C).

Under the less stringent definition of the flanking regions [-1500, +500] bp, three additional clusters (Clusters 4-6) were found enriched for SRF in the HRG time-course. The three clusters are characterized by different expression patterns (Clusters 4-5: intermediate expression pattern; Cluster 6: late expression pattern, Supplementary Figure S6B-C). The SRF-enriched Cluster 4 and its subcluster Cl. 5 group genes with an intermediate response pattern consistent with the slow increase in the SRF protein levels (Figure 2B). Clusters 4-5 include 205 promoters involved in *cell death* and *response to growth factor*. Within Cluster 4, Cluster 5 isolates a subset of 65 promoters involved in *angiogenesis/blood vessel development*. Finally, Cluster 6 is a collection of 20 late response promoters, whose expression slowly increases and peaks at 6-8 hours (Supplementary Figure S6C). Consistently with these clusters, SRF CAGE expression dynamics (Figure 2A) and SRF

protein dynamics (Figure 2B) are characterized by a double peaks pattern, respectively at 30 min and 3 hours, suggesting that SRF plays multiple roles in the response to HRG stimuli.

REFERENCES

1. Mina, M., Jurman, G. and Furlanello, C. CIDER: a pipeline for detecting waves of coordinated transcriptional regulation in gene expression time-course data. *bioRxiv* (2015).
2. Iacono,G., Altafini,C. and Torre,V. Early phase of plasticity-related gene regulation and SRF dependent transcription in the hippocampus. *PloS One* **8,** e68078 (2013).
3. Dijkmans,T.F., van Hooijdonk,L.W., Schouten,T.G., Kamphorst,J.T., Fitzsimons,C.P. and Vreugdenhil,E. Identification of new nerve growth factor-responsive immediate-early genes. *Brain Research* **1249,** 19–33 (2009).
4. Muller,M., *Information Retrieval for Music and Motion* **Ch. 4,** Springer, ISBN 978-3-540-74047-6 (2007).
5. Furlanello,C., Merler,S. and Jurman,G. Combining feature selection and DTW for time-varying functional genomics. *IEEE Transactions on Signal Processing* **54,** 2436-2443 (2006).
6. Sakoe,H and Chiba,S. Dynamic programming algorithm optimization for spoken word recognition. *IEEE Transactions on Acoustics, Speech and Signal Processing* **26,** 43-49 (1978).
7. Batista,G.E.A.P.A., Keogh,E.J., Tataw,O.M. and Souza,V.M.A.d.S. CID: an efficient complexity-invariant distance for time series. *Data Mining and Knowledge Discovery* **28,** 634–669 (2014).
8. Giorgino,T. Computing and Visualizing Dynamic Time Warping Alignments in R: The dtw Package. *Journal of Statistical Software* **31,** 7 (2009).
9. Müllner,D. fastcluster: Fast Hierarchical, Agglomerative Clustering Routines for R and Python. *Journal of Statistical Software* **53,** 9 (2013).
10. McLeay, R.C. and Bailey,T.L. Motif Enrichment Analysis: a unified framework and an evaluation on ChIP data. *BMC bioinformatics* **11,** 165 (2010).
11. Tange, O. GNU Parallel - The Command-Line Power Tool. *The USENIX Magazine*, 42-47 (2011).
12. Mathelier,A. et al. JASPAR 2014: an extensively expanded and updated open-access database of transcription factor binding profiles. *Nucleic Acids Research* **42,** D142-7 (2014).
13. Li,H., Handsaker,B., Wysoker,A., Fennell,T., Ruan,J., Homer,N., Marth,J. and Durbin,R. The Sequence Alignment/Map format and SAMtools. *Bioinformatics* **25,** 2078–9 (2009).
14. Arner, E., Daub, C.O., Vitting-Seerup, K., Andersson, R., Lilje, B., Drabløs, F., et al. Transcribed enhancers lead waves of coordinated transcription in transitioning mammalian cells. *Science* **347**, 1010-1014 (2015).

15. Veerla, S., Ringnér, M. and Höglund, M. Genome-wide transcription factor binding site/promoter databases for the analysis of gene sets and co-occurrence of transcription factor binding motifs. *BMC genomics* **11,** 145 (2010).

16. Worsley-Hunt,R., Mathelier,A., Del Peso,L. and Wasserman,W.W. Improving analysis of transcription factor binding sites within ChIP-Seq data based on topological motif enrichment. *BMC Genomics* **15,** 472 (2014).

17. Sui,S.J.H, Fulton,D.L., Arenillas,D.J., Kwon,A.T. and Wasserman,W.W. oPOSSUM: integrated tools for analysis of regulatory motif over-representation. *Nucleic Acids Research* **35,** W245–52 (2007).

18. Sui,S.J.H., Mortimer,J.R., Arenillas,D.J., Brumm,J., Walsh,C.J., Kennedy,B.P. and Wasserman,W.W. oPOSSUM: identification of over-represented transcription factor binding sites in co-expressed genes. *Nucleic Acids Research* **33,** 3154–64 (2005).

19. Kwon,A.T., Arenillas,D.J., Hunt,R.W. and Wasserman,W.W. oPOSSUM-3: advanced analysis of regulatory motif over-representation across genes or ChIP-Seq datasets. *G3* **2,** 987–1002 (2012).

20. The FANTOM Consortium & Riken Omics Science Center The transcriptional network that controls growth arrest and differentiation in a human myeloid leukemia cell line. *Nature Genetics* **41,** 553–62 (2009).

CONSORTIA

$ List of The FANTOM Consortium members by affiliation – participants to the project should not be considered co-authors:

- Anatomy and Embryology, Leiden University Medical Center, Einthovenweg 20, P.O. Box 9600, 2300 RC Leiden, The Netherlands.

Christine MUMMERY, Robert PASSIER

- Australian Infectious Diseases Research Centre (AID), University of Queensland, Brisbane St Lucia, QLD 4072, Australia.

Anthony BECKHOUSE, Antje Blumenthal, Christine WELLS, Dipti VIJAYAN, Kelly HITCHENS

- Australian Institute for Bioengineering and Nanotechnology (AIBN), University of Queensland, Brisbane St Lucia, QLD 4072, Australia.

Anthony BECKHOUSE, Christine WELLS, Dipti VIJAYAN, Dmitry OVCHINNIKOV, Ernst WOLVETANG, James BRIGGS, Kelly HITCHENS

- Bioinformatics and Computational Biology, The Jackson Laboratory, 600 Main Street, Bar Harbor, ME 04609 USA.

Judith A. BLAKE

- Biological and Environmental Sciences and Engineering Division, King Abdullah University of Science and Technology (KAUST), Ibn Al-Haytham Building -2, Thuwal 23955-6900, Kingdom of Saudi Arabia.

Carlo CANNISTRACI, Timothy RAVASI

- Biozentrum, University of Basel, Klingelbergstrasse 50-70, 4056 Basel, Switzerland.

Erik VAN NIMWEGEN, Piotr BALWIERZ

- Cancer Biology Program, Mater Medical Research Institute, Raymond Tce, South Brisbane, QLD 4101, Australia.

Geoffery FAULKNER

- Center for Clinical and Translational Reseach, Kyushu University Hospital, Station for Collaborative Research1 4F, 3-1-1 Maidashi, Higashi-Ku, Fukuoka, 812-8582 Japan.

Daisuke SUGIYAMA

- Center for Molecular Medicine and Genetics, Wayne State University, 3228 Scott Hall, 540 East Canfield Street, Detroit, MI 48201-1928, USA.

Emily J. WOOD, Hui JIA, Leonard LIPOVICH

- Center for Radioisotope Sciences, Tohoku University Graduate School of Medicine, 2-1 Seiryo-machi, Aoba-ku, Sendai, Miyagi, 980-8575 Japan.

Hozumi MOTOHASHI

- Centre for Vascular Research, University of New South Wales, Sydney NSW 2052, Australia.

Levon KHACHIGIAN

- Columbia Initiative in Systems Biology, Columbia University Medical Center, Herbert Irving Comprehensive Cancer Center, 1130 St. Nicholas Ave, New York, NY 10032 USA.

Yishai SHIMONI

- Computational Biology Research Center, National Institute of Advanced Industrial Science and Technology (AIST), 2-4-7 Aomi, Koto-ku, Tokyo, 135-0064 Japan.

Martin FRITH

- Computational Bioscience Research Center, King Abdullah University of Science and Technology (KAUST), Ibn Al-Haytham Building -2, Thuwal 23955-6900, Kingdom of Saudi Arabia.

Adam BALIC, Arnab PAIN, Benoit MARCHAND, Boris JANKOVIC, Intikhab ALAM, John ARCHER, Mamoon RASHID, Sebastian SCHMEIER, Ulf SCHAEFER, Vladmir BAJIC, Yulia MEDVEDEVA

- Department of Biochemistry and Cell Biology, Rice University, Houston, Texas, USA.

Mary C. FARACH-CARSON

- Department of Biochemistry and Mol. Biophysics, Columbia University Medical Center, 701 West 168th Street, New York, NY 10032 USA.

Andrea CALIFANO

- Department of Biochemistry, Ohu University School of Pharmaceutical Sciences, Misumido 31-1, Tomitamachi, Koriyama, Fukushima, 963-8611 Japan.

Mitsuhiro OHSHIMA

- Department of Bioinformatics, Medical Research Institute, Tokyo Medical and Dental University, 1-5-45 Yushima, Bunkyo-ku, Tokyo, 113-8510 Japan.

Hiroshi TANAKA, Soichi OGISHIMA

- Department of Biological Sciences, University of Delaware, Newark, Delaware, USA.

Swati PRADHAN-BHATT

- Department of Biology, University of Bergen, Thormøhlensgate 53, NO-5006 Bergen, Norway.

Vanja HABERLE

- Department of Biosciences and Nutrition, Center for Biosciences, Karolinska Institutet, Hälsovägen 7-9, SE-141 83 Huddinge, Sweden.

Andreas LENNARTSSON, Helena PERSSON, Juha KERE, Karl EKWALL

- Department of Biostatistics, Harvard School of Public Health, 655 Huntington Ave, Boston, MA 02115, USA.

Emmanuel DIMONT, Gabriel ALTSCHULER, Oliver HOFMANN, Shannan HO SUI, Winston HIDE

- Department of Cancer Research and Molecular Medicine, Norwegian University of Science and Technology (NTNU), P.O. Box 8905, NO-7491 Trondheim, Norway.

Finn DRABROS, Morten RYE

- Department of Cell and Molecular Biology, Karolinska Institutet, P.O. Box 285, SE-171 77 Stockholm, Sweden.

Lukasz HUMINIECKI, Morana VITEZIC

- Department of Clinical Genetics, VU University Medical Center Amsterdam, Van der Boechorststraat 7, 1081 BT Amsterdam, The Netherlands.

Margherita FRANCESCATTO, Patrizia RIZZU, Peter HEUTINK

- Department of Clinical Molecular Genetics, School of Pharmacy, Tokyo University of Pharmacy and Life Sciences, 1432-1 Horinouchi, Hachioji, Tokyo 192-0392, Japan.

Hiroo TOYODA, Tadasuke NOZAKI

- Department of Computational Systems Biology, Vavilov Institute of General Genetics (VIGG), Gubkin str. 3, Moscow 119991, Russia.

Alexander FAVOROV, Artem KASIANOV, Ilya VORONTZOV, Ivan KULAKOVSKIY, Vsevolod MAKEEV

- Department of Computer Science, University of Bristol, Merchant Venturers Building, Woodland Road, Clifton BS8 1UB, UK.

Hai FANG, Julian GOUGH, Owen RACKHAM

- Department of Dermatology and Allergy, Charité Campus Mitte, Universitätsmedizin Berlin, Chariteplatz 1, 10117 Berlin, Germany.

Magda BABINA, Sven GUHL

- Department of Hematopoietic Factor, Institute of Medical Science, University of Tokyo, Tokyo 108-8639, Japan.

Fumio NAKAHARA, Toshio KITAMURA

- Department of Human Genetics, Leiden University Medical Center, Einthovenweg 20, 2333 ZC Leiden, The Netherlands.

Andrew GIBSON, Erik A SCHULTES, Jeroen LAROS, Mark THOMPSON, Peter T Hoen, Zuotian TATUM

- Department of Informatics, University of Bergen, Høgteknologisenteret, Thormøhlensgate 53, NO-5008 Bergen, Norway.

Boris LENHARD

- Department of Internal Medicine III, University Hospital Regensburg, F.-J.-Strauss Allee 11, D-93042 Regensburg, Germany.

Christian SCHMIDL, Matthias EDINGER, Michael REHLI

- Department of Medical Biochemistry, Tohoku University Graduate School of Medicine, 2-1 Seiryo-machi, Aoba-ku, Sendai, Miyagi, 980-8575 Japan.

Hironori SATOH, Jun TAKAI, Masayuki YAMAMOTO, Rie FUJITA

- Department of Medical Genetics, Centre for Molecular Medicine and Therapeutics, Child and Family Research Institute , University of British Columbia , 950 West 28th Avenue, Vancouver, BC V5Z 4H4, Canada.

Anthony MATHELIER, Daniel GOLDOWITZ, Peter ZHANG, Thomas HA, Wyeth WASSERMAN

- Department of Medicine, Karolinska Institutet at Karolinska University Hospital, Huddinge, SE-141 86 Huddinge, Sweden.

Niklas MEJHERT, Peter ARNER

- Department of Microbiology and Immunology, Keio University School of Medicine, 35 Shinanomachi, Shinjuku, Tokyo, 160-8582 Japan.

Jun-ichi FURUSAWA, Kazuyo MORO, Shigeo KOYASU

- Department of Molecular and Cellular Biology, Harvard University, 16 Divinity Ave, Cambridge, MA 02138, USA.

Eivind VALEN, Yoko YAMAGUCHI

- Department of Neurology, University at Buffalo School of Medicine and Biomedical Sciences, New York State Center of Excellence in Bioinformatics and Life Sciences, 701 Ellicott Street, Buffalo, NY 14203 USA.

Alexander DIEHL

- Department of Orthopedic, Trauma and Reconstructive Surgery, Charité Universitätsmedizin Berlin, Garystrasse 5, 14195 Berlin, Germany.

Gundula SCHULZE-TANZIL

- Department of Otology and Laryngology, Harvard Medical School, Massachusetts Eye and Ear Infirmary, Eaton-Peabody Lab, 243 Charles Street, Boston, MA 02114, USA.

Albert EDGE, Judith KEMPFLE

- Diamantina Institute, University of Queensland, Brisbane St Lucia, QLD 4072, Australia.

Antje Blumenthal, Tony KENNA

- Division of Functional Genomics and Systems Medicine, Research Center for Genomic Medicine, Saitama Medical University, 1397-1 Yamane, Hidaka, Saitama, 350-1241 Japan.

Yasushi OKAZAKI, Yosuke MIZUNO, Yutaka NAKACHI

- Division of Immunology, Institute of Infectious Diseases and Molecular Medicine (IIDMM), University of Cape Town, Anzio Road, Observatory 7925, Cape Town, South Africa.

Anita SCHWEGMANN, Frank BROMBACHER, Reto GULER, Suzana SAVVI

- Engelhardt Institute of Molecular Biology, Russian Academy of Sciences, Vavilov str. 32, Moscow 119991, Russia.

Ivan KULAKOVSKIY, Vsevolod MAKEEV

- Experimental Immunology, Academic Medical Center - University of Amsterdam, Meibergdreef 9 , 1105 AZ Amsterdam, The Netherlands.

Linda VAN DEN BERG, Peter T Hoen, Teunis BH GEIJTENBEEK

- Experimental Immunology, Immunology Frontier Research Center, Osaka University, 3-1 Yamadaoka, Suita, Osaka, 565-0871 Japan.

Hiromasa MORIKAWA, Masahide HAMAGUCHI, Naganari OHKURA, Shimon SAKAGUCHI

- Faculty of Engineering, University of Bristol, Merchant Venturers Building, Woodland Road, Clifton BS8 1UB, UK.

David DE LIMA MORAIS

- Faculty of Medicine, Institute of Clinical Sciences, MRC Clinical Sciences Centre, Imperial College London, Hammersmith Hospital Campus, London W12 0NN, UK.

Vanja HABERLE, Boris LENHARD

- FM Kirby Neurobiology Center, Children’s Hospital Boston, Harvard . Medical School, 300 Longwood Ave, Boston MA 02115, USA.

Michela FAGIOLINI

- Fondazione Santa Lucia, Dulbecco Telethon Institute, via del Fosso di Fiorano 64, 00143 Rome RM, Italy.

Beatrice BODEGA, Valerio ORLANDO

- Gastroenterology, Research Center for Hepatitis and Immunology Research Institute, National Center for Global Health and Medicine, 1-7-1 Kohnodai, Ichikawa, Chiba, 272-8516 Japan.

Taeko DOHI, Yuki KAWAMURA

- Genomics Division, Lawrence Berkeley National Laboratory, 84R01, 1 Cyclotron Road, Berkeley, CA 94720 USA.

Christopher J. MUNGALL

- Genomics, Cold Spring Harbor Laboratory, 1 Bungtown Road, Cold Spring Harbor , NY 11797, USA.

Carrie DAVIS, Thomas GINGERAS

- Graduate Program in Areas of Basic and Applied Biology, Abel Salazar Biomedical Sciences Institute, University of Porto, Rua de Jorge Viterbo Ferreira n. 228, 4050-313 Porto, Portugal.

Margherita FRANCESCATTO

- Graduate School of Pharmaceutical Sciences, Nagoya University, Furo-cho, Chikusa, Nagoya, Aichi, 464-8601 Japan.

Hideki TATSUKAWA

- Harry Perkins Institute of Medical Research, and the Centre for Medical Research, University of Western Australia. QQ Block, QEII Medical Centre, Nedlands, Perth, WA 6009, Australia.

Louise WINTERINGHAM, Peter KLINKEN

- Hjelt Institute, Department of Forensic Medicine, University of Helsinki, Kytosuontie 11, 003000 Helsinki, Finland.

Antti SAJANTILA

- Hubrecht Institute, Uppsalalaan 8, 3584 CT Utrecht, The Netherlands.

Hans CLEVERS, Marc VAN DE WETERING

- Immunology and Infectious Disease, International Centre for Genetic Engineering & Biotechnology (ICGEB) Cape Town component, Anzio Road, Observatory 7925, Cape Town, South Africa.

Anita SCHWEGMANN, Frank BROMBACHER, Reto GULER, Suzana SAVVI

- Institute of Pharmaceutical Sciences, ETH Zurich, Wolfgang-Pauli-Strasse 10, HCI H 303, 8093 Zurich, Switzerland.

Michael DETMAR, Sarah KRAMPITZ

- Laboratorio Nazionale del Consorzio Interuniversitario per le Biotecnologie (LNCIB), Padriciano 99, 34149 Trieste TS, Italy.

Claudio SCHNEIDER, Emiliano DALLA, Roberto VERARDO, Silvano PIAZZA, Yari CIANI

- Laboratory Animal Research Center, Institute of Medical Science, The University of Tokyo, 4-6-1 Shirokanedai, Minato-ku, Tokyo, 108-8639 Japan.

Chieko KAI, Hiroki SATO, Misako YONEDA, Takaaki SUGIYAMA, Toshiyuki NAKAMURA

- Melanoma Research Center, The Wistar Institute, 3601 Spruce Street, Philadelphia, PA 19104, USA.

Meenhard HERLYN, Rolf SWOBODA

- Molecular and Cellular Oncogenesis, The Wistar Institute, 3601 Spruce Street, Philadelphia, PA 19104, USA.

Suzan E. ZABIEROWSKI

- Mouse Informatics, European Molecular Biology Laboratory, European Bioinformatics Institute, Wellcome Trust Genome Campus, Hinxton, Cambridge, CB10 1SD, UK.

Terrence F. MEEHAN

- MRC Human Genetics Unit, MRC Institute of Genetics and Molecular Medicine (MRC-IGMM), University of Edinburgh, Western General Hospital, Crewe Road, Edinburgh, EH4 2XU, UK.

Alison MEYNERT, Colin SEMPLE, James PRENDERGAST, Martin TAYLOR, Robert Young, Sarah BAKER

- National Centre for Adult Stem Cell Research, Eskitis Institute for Cell and Molecular Therapies, Griffith University, Brisbane, Queensland, Australia.

Alan MACKAY-SIM

- Neuroscience, SISSA, via Bonomea 265, 34136 Trieste TS, Italy.

Silvia ZUCCHELLI, Stefano GUSTINCICH

- Predictive Models for Biomedicine and Environment, Fondazione Bruno Kessler, via Sommarive 18, 38123 Trento TN, Italy.

Cesare FURLANELLO, Davide ALBANESE, Giuseppe JURMAN, Marco CHIERICI, Marco RONCADOR

- Respiratory Medicine, University of Nottingham, Clinical Sciences Building, City Hospital, Hucknall Road, Nottingham, NG5 1PB, UK.

Alan KNOX

- RIKEN Advanced Science Institute (ASI), 2-1 Hirosawa, Wako, Saitama, 351-0198 Japan.

Mitsuko HARA, Soichi KOJIMA

- RIKEN Bioinformatics And Systems Engineering Division (BASE), 1-7-22 Suehiro, Tsurumi, Yokohama, Kanagawa, 230-0045, Japan.

Kei IIDA, Shuji KAWAGUCHI, Tetsuro TOYODA

- RIKEN BioResource Center (BRC), Koyadai 3-1-1, Tsukuba, Ibaraki, 305-0074 Japan.

Yukio NAKAMURA

- RIKEN Center for Developmental Biology (CDB), 2-2-3 Minatojima-minamimachi, Chuo-ku, Kobe, Hyogo, 650-0047 Japan.

Guojun SHENG, Hideki ENOMOTO, Mitsuru MORIMOTO, Yohei YONEKURA

- RIKEN Center for Life Science Technologies (Division of Genomic Technologies), 1-7-22 Suehiro-cho, Tsurumi-ku, Yokohama, 230-0045 Japan.

Takeya KASUKAWA, Akira HASEGAWA, Alessandro BONETTI, Alistair FORREST, Andrew Tae Jun KWON, Bogumil Kaczkowski, Charles PLESSY, Christophe SIMON, Efthymios MOTAKIS, Erik ARNER, Fumi HORI, Harukazu SUZUKI, Hiroko OHMIYA, Hiromi NISHIYORI (SUEKI), Jay SHIN, Jayson HARSHBARGER, Jessica SEVERIN, Jordan RAMILOWSKI, Kaoru KAIDA, Kazuhiro KAJIYAMA, Marina LIZIO, Masaaki FURUNO, Michiel DE HOON, Michihira TAGAMI, Miki KOJIMA, Mitsuyoshi MURATA, Mizuho SAKAI, Naoko SUZUKI, Naoko TAKAHASHI, Naoto KONDO, Nicolas BERTIN, Piero CARNINCI, Riichiro MANABE, Sachi KATO (ISHIKAWA), Shigehiro YOSHIDA, Shohei NOMA, Sugata ROY, Takahiro ARAKAWA, Timo LASSMANN, Tsugumi KAWASHIMA, Yuki HASEGAWA, Yuri ISHIZU

- RIKEN Omics Science Center (OSC), 1-7-22 Suehiro-cho, Tsurumi-ku, Yokohama, 230-0045 Japan.

Ai KAIHO, Akiko SAKA, Akira HASEGAWA, Alessandro BONETTI, Alistair FORREST, Alka SAXENA, Andrew Tae Jun KWON, Atsutaka KUBOSAKI, Bogumil Kaczkowski, Carsten DAUB, Charles PLESSY, Christophe SIMON, Efthymios MOTAKIS, Eri SAIJYO, Erik ARNER, Fumi HORI, Harukazu SUZUKI, Hideya KAWAJI, Hiroko OHMIYA, Hiromi NISHIYORI (SUEKI), Hisashi SHIMOJI, Jay SHIN, Jayson HARSHBARGER, Jessica SEVERIN, Jordan RAMILOWSKI, Jun KAWAI, Kaoru KAIDA, Kazuhiro KAJIYAMA, Kenichi NAKAZATO, Marina LIZIO, Masaaki FURUNO, Masanori SUZUKI, Masayoshi ITOH, Matthias HARBERS, Max BURROUGHS, Michiel DE HOON, Michihira TAGAMI, Miki KOJIMA, Mitsuyoshi MURATA, Mizuho SAKAI, Morana VITEZIC, Mutsuni KANAMORI-KATAYAMA, Naoko SUZUKI, Naoko TAKAHASHI, Naoto KONDO, Nicolas BERTIN, Noriko

NINOMIYA (FUKUDA), Piero CARNINCI, Riichiro MANABE, Sachi KATO (ISHIKAWA), Sayaka NAGAO (SATO), Shigehiro YOSHIDA, Shintaro KATAYAMA, Shiro FUKUDA, Shohei NOMA, Shoko WATANABE, Sugata ROY, Takahiro ARAKAWA, Takehiro HASHIMOTO, Thierry SENGSTAG, Timo LASSMANN, Tsugumi KAWASHIMA, Yoshihide HAYASHIZAKI, Yuki HASEGAWA, Yuri ISHIZU

- RIKEN Preventive Medicine and Diagnosis Innovation Program, 1-7-22 Suehiro-cho, Tsurumi-ku, Yokohama, 230-0045 Japan.

Hideya KAWAJI, Jun KAWAI, Masayoshi ITOH, Yoshihide HAYASHIZAKI

- RIKEN Research Center for Allergy and Immunology (RCAI), 1-7-22 Suehiro, Tsurumi, Yokohama, Kanagawa, 230-0045, Japan.

Haruhiko KOSEKI, Hiroshi KAWAMOTO, Hiroshi OHNO, Jun-ichi FURUSAWA, Kazuyo MORO, Mariko OKADA-HATAKEYAMA, Mitsuhiro ENDOH, Shigeo KOYASU, Tomokatsu IKAWA

- Science for Life Laboratory, Box 1031, SE-171 21 Solna, Sweden.

Juha KERE

- Systems and Computational Biology, Albert Einstein College of Medicine, 1300 Morris Park Ave Price 253, New York, NY 10461, USA.

Daniel CARBAJO, Jessica MAR

- The Bioinformatics Centre, Department of Biology and BRIC, University of Copenhagen, Ole Maaloes Vej 5, DK 2200 Copenhagen, Denmark

Albin SANDELIN, Berit LILJE, Ilka HOOF, Kang LI, Mette JORGENSEN, Robin ANDERSSON, Xiaobei ZHAO, Yun CHEN

- The Roslin Institute and Royal (Dick) School of Veterinary Studies, University of Edinburgh, Easter Bush, Edinburgh, Midlothian, EH25 9RG Scotland, UK.

Anagha JOSHI, David HUME, Kenneth BAILLIE, Kim SUMMERS, Lynsey FAIRBAIRN, Malcolm FISHER, Tom FREEMAN
